# scATACpipe: A nextflow pipeline for comprehensive and reproducible analyses of single cell ATAC-seq data

**DOI:** 10.3389/fcell.2022.981859

**Published:** 2022-09-27

**Authors:** Kai Hu, Haibo Liu, Nathan D. Lawson, Lihua Julie Zhu

**Affiliations:** ^1^ Department of Molecular, Cell and Cancer Biology, University of Massachusetts Chan Medical School, Worcester, MA, United States; ^2^ Program in Molecular Medicine, Program in Bioinformatics and Integrative Biology, University of Massachusetts Chan Medical School, Worcester, MA, United States

**Keywords:** scATAC-seq, chromatin accessibility, single cell, nextflow, pipeline, transcription factor activity and footprinting analysis, integration of scATAC-seq and scRNA-seq, trajectory inference

## Abstract

Single cell ATAC-seq (scATAC-seq) has become the most widely used method for profiling open chromatin landscape of heterogeneous cell populations at a single-cell resolution. Although numerous software tools and pipelines have been developed, an easy-to-use, scalable, reproducible, and comprehensive pipeline for scATAC-seq data analyses is still lacking. To fill this gap, we developed scATACpipe, a Nextflow pipeline, for performing comprehensive analyses of scATAC-seq data including extensive quality assessment, preprocessing, dimension reduction, clustering, peak calling, differential accessibility inference, integration with scRNA-seq data, transcription factor activity and footprinting analysis, co-accessibility inference, and cell trajectory prediction. scATACpipe enables users to perform the end-to-end analysis of scATAC-seq data with three sub-workflow options for preprocessing that leverage 10x Genomics Cell Ranger ATAC software, the ultra-fast Chromap procedures, and a set of custom scripts implementing current best practices for scATAC-seq data preprocessing. The pipeline extends the R package ArchR for downstream analysis with added support to any eukaryotic species with an annotated reference genome. Importantly, scATACpipe generates an all-in-one HTML report for the entire analysis and outputs cluster-specific BAM, BED, and BigWig files for visualization in a genome browser. scATACpipe eliminates the need for users to chain different tools together and facilitates reproducible and comprehensive analyses of scATAC-seq data from raw reads to various biological insights with minimal changes of configuration settings for different computing environments or species. By applying it to public datasets, we illustrated the utility, flexibility, versatility, and reliability of our pipeline, and demonstrated that our scATACpipe outperforms other workflows.

## 1 Introduction

Cell heterogeneity is a universal phenomenon in living organisms (Altschuler and Wu, 2010; Martins and Locke, 2015; Goldman et al., 2019) and even in seemingly pure cell lines cultured *in vitro* (Hastings and Franks, 1983; Toyooka et al., 2008; Sato et al., 2016; SoRelle et al., 2021), intrinsically contributing to tissue diversity and functionality. As cells are the fundamental building blocks of multicellular organisms, it is crucial to understand the mechanisms that control cell heterogeneity. Besides diverse and delicate internal and external cues, cell heterogeneity is largely controlled by differences in gene expression, which are orchestrated by intricate interactions among diverse *trans*-acting factors, including transcription factors and chromatin remodelers, and *cis*-regulatory elements (CREs), such as promoters, enhancers, and insulators, which are interspersed throughout the genome (Carter and Zhao, 2021). Large-scale studies have shown that a majority of such functional CREs are located in open chromatin regions, which are nucleosome-depleted and thus accessible to *trans*-acting factors (The ENCODE Consortium, 2019). Single cell ATAC-seq (scATAC-seq) (Buenrostro et al., 2015; Cusanovich et al., 2015), a recent innovative combination of the ATAC-seq (Assay for Transposase-Accessible Chromatin using sequencing) method (Buenrostro et al., 2013) and single cell technologies (Li and Humphreys, 2021), is currently the most widely used approach for profiling the genome-wide landscape of open chromatin regions at the single-cell level. An in-depth analysis of scATAC-seq data can reveal distinct cell populations, roles of key transcription factor, gene regulatory programs underlying cell heterogeneity, and trajectories of cell lineage differentiation (Baek and Lee, 2020; Granja et al., 2021). So far, scATAC-seq has been used for investigating epigenetic heterogeneity in complex tissues during normal development and diseases, such as an array of adult tissues (Cusanovich et al., 2018; Liu et al., 2019; Zhang K. et al., 2021; Chen et al., 2021; Fang et al., 2021), developing tissues and embryos (Preissl et al., 2018; Pijuan-Sala et al., 2020), immune cell development (Buenrostro et al., 2018; Satpathy et al., 2019), spermatogenesis (Wu et al., 2021), and tumor progression (LaFave et al., 2020; Taavitsainen et al., 2021).

Over the past 6 years, different scATAC-seq technologies have been developed with various throughput, including nanowell-based (TaKaRa ICELL8 system) (Mezger et al., 2018), circuit microfluidics-based (Fluidigm C1 system) (Buenrostro et al., 2015), droplet microfluidics-based (10x Genomics Chromium system) (Zheng et al., 2017), split-pool combinatorial indexing-based (Cusanovich et al., 2015), and more recent droplet-based combinatorial indexing ATAC-seq technologies (Lareau et al., 2019). However, data generated by all these different technologies are intrinsically very noisy, sparse, and high dimensional (Chen et al., 2019), which makes it challenging to obtain biological insights from the raw sequencing data (Chen et al., 2019; Fang et al., 2021). To date, more than a dozen of software tools, such as ArchR (Granja et al., 2021), SnapATAC (Fang et al., 2021), and Signac (Stuart et al., 2020), and a few pipelines, such as scATAC-pro (Yu et al., 2020) and MAESTRO (Wang et al., 2020), have been adopted or specifically developed for scATAC-seq data analyses. Some of the tools have been benchmarked (Chen et al., 2019) and some best practices have been established (Chen et al., 2019; Baek and Lee, 2020; Yu et al., 2020). In general, each tool has a subset of functionalities, and a chain of tools are needed for a comprehensive analysis of the scATAC-seq data. We summarized the properties of existing tools in Supplementary Table S1. In short, only workflow-based approaches support end-to-end analysis. However, none of them can handle large datasets with millions of cells or support data analysis for species other than the human and the mouse. Thus, what is still lacking is easy-to-use, reliable, reproducible, and comprehensive pipelines that integrate all best practices and functionalities.

To fill this gap, we developed a scalable and robust pipeline called scATACpipe (https://github.com/hukai916/scATACpipe) for analyzing scATAC-seq data with a comprehensive set of functionalities including raw sequencing data quality control (QC), adaptor trimming, barcode correction, debarcoding, read alignment, alignment file manipulation, global and cell-level post-alignment QC and filtering, annotation file preparation, feature-by-cell matrix formation, batch correction, dimension reduction, visualization, clustering, integration with scRNA-seq data, cell identity annotation, differential accessibility analysis, transcription factor activity inference and footprinting analysis, gene activity prediction, co-accessibility inference, and cellular trajectory analysis. scATACpipe can be easily adapted to data generated on most single-cell platforms. The pipeline is powered by a state-of-art workflow management engine, Nextflow (Di Tommaso et al., 2017), making it easy to be deployed to a variety of computing environments. In short, scATACpipe allows reproducible and comprehensive analysis of scATAC-seq data from raw reads to various biological insights. In this paper, we demonstrate the application of scATACpipe using public scATAC-seq datasets and its superior performance by comparing it with other two major pipelines for scATAC-seq data analysis.

## 2 Methods and materials

### 2.1 Implementation

Powered by Nextflow, which was developed with a strong focus on portability, reproducibility, scalability, and usability (Di Tommaso et al., 2017), scATACpipe integrates many carefully selected open-source software tools and custom scripts written in R, Python, or Bash for a comprehensive analysis of scATAC-seq data with up to millions of cells. Detailed information about the software adopted in the customized preprocessing workflow is shown in Supplementary Table S2. In accordance with the best practices of Nextflow, individual major processes focusing on specific tasks are modularized in scATACpipe. While the custom scripts are available as built-in workflow components, all third-party software dependencies are built into individual Docker images hosted in the Docker Hub (Merkel, 2014; Kurtzer et al., 2017), and these Docker images can be automatically converted into Singularity images if Singularity is set as the execution environment. With minimal modifications of the configuration files and/or command-line parameter settings, the pipeline can be run on a local computer with a Unix-like OS, such as Linux and Mac OS, a high-performance computing cluster, or a cloud computing environment. Users can find detailed instructions for setting up the running environment in the usage documentation (https://github.com/hukai916/scATACpipe/blob/main/docs/usage.md#custom-configuration). To facilitate the preparation of the configuration file, we have also implemented a web application (https://mccb.umassmed.edu/scATACpipe/ConfigGenerator/index.html) for users to interactively set parameters for running the pipeline in different computing environments. Users can also download the application (https://github.com/hukai916/scATACpipe#web-gui) and run it locally.

**FIGURE 1:**
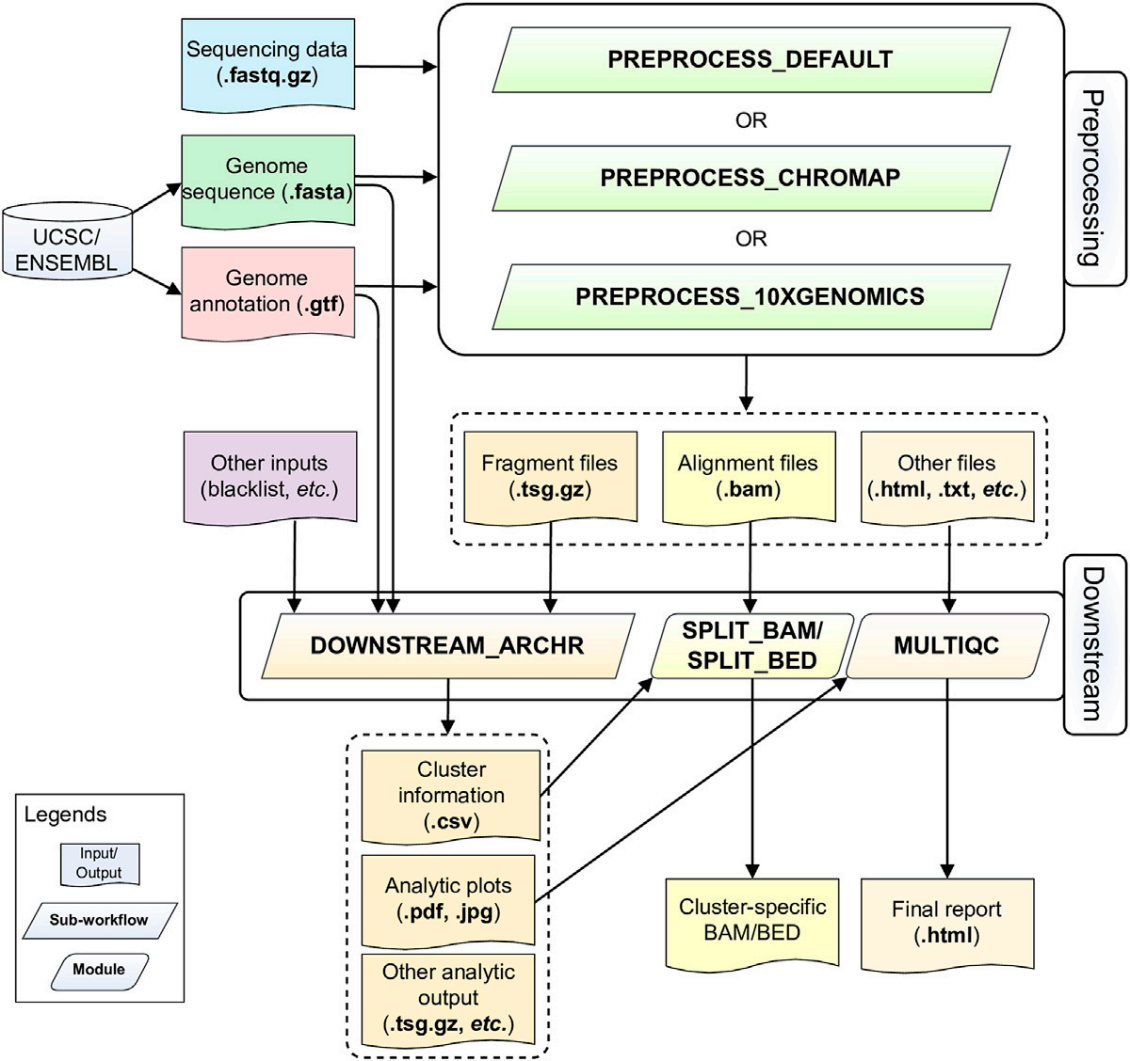
Schematic diagram of the scATACpipe workflow. The scATACpipe workflow is mainly composed of modules for the preprocessing sub-workflow and the downstream analysis sub-workflow. The preprocessing sub-workflow offers three options: the 10x Genomics Cell Ranger ATAC software-based preprocessing, the ultra-fast Chromap-based preprocessing, and the default preprocessing which implements the current best practices for scATAC-seq data analysis. The preprocessing sub-workflow takes sequencing data (.fastq.gz files), a reference genome sequence (.fasta file), and a genome annotation file (.gtf file) as inputs. The last two files can be automatically downloaded from the UCSC or Ensembl Genome Browser. Alternatively, users can specify a genome index compatible with the mapper of the desired preprocessing option. The fragment file generated from the preprocessing sub-workflow is passed along with an optional genome blacklist file and other custom inputs to the downstream analysis sub-workflow for core analyses, including batch correction, clustering, integration with scRNA-seq data, cluster annotation, peak calling, marker gene and marker peak identification, motif enrichment and deviation analysis, footprinting analysis, integrated analysis, and trajectory analysis using mainly the ArchR package. The alignment file (.bam) and fragment file (.tsv.gz) from the preprocessing sub-workflow are converted into cluster-specific BAM, BED, and/or BigWig files for visualization in genome browsers. An interactive HTML report is generated at the end with all analytic plots from the downstream analyses, and QC summaries from the default preprocessing sub-workflow if chosen.

ScATACpipe consists of two major groups of functional modules, one for preprocessing scATAC-seq data from fastq files to fragment files, and the other for downstream analysis (Figure 1). Currently, scATACpipe provides three options for data preprocessing: a default customized preprocessing sub-workflow, the 10x Genomics Cell Ranger ATAC software-based preprocessing sub-workflow, and the Chromap-based (Zhang H. et al., 2021) preprocessing sub-workflow. The pipeline uses mainly the R package ArchR (Granja et al., 2021) for downstream analysis, which is the only tool that can handle scATAC-seq data of millions of cells. Importantly, with the help of our custom scripts, scATACpipe can process scATAC-seq data from any eukaryotic species with an annotated reference genome though ArchR currently only supports four genome assemblies (human hg19 and hg38, mouse mm9 and mm10) natively. In addition, a comprehensive HTML report for both default preprocessing and the entire downstream analysis is provided for easy navigation and visualization. Furthermore, cluster-specific BED, BAM and BigWig files are generated for visualization in genome browsers.

Users can start their analyses by providing a sample sheet in the CSV format, which specifies sample names, absolute paths to fastq files of paired-end reads for genomic DNA inserts and cell barcodes, and choosing one of the three preprocessing sub-workflows. Subsequently, the downstream analysis is performed using the ArchR package-based sub-workflow. Alternatively, users can directly start the downstream analysis with bgzip-compressed fragment files. With our pipeline, users can easily rerun part of the analysis with fine-tuned parameters by setting the command line option -*resume*. With this option, only modules affected by updated parameters are rerun. As a result, the pipeline can be efficiently executed multiple times to achieve desired outcomes.

### 2.2 Description of scATACpipe modules

Each major task in scATACpipe has been modularized in accordance with the best practices suggested by the Nextflow community. This resulted in 89 individual modules that are distributed across six sub-workflows: three for preprocessing, one for downstream analysis, and two for input file validation. The name, incorporated software, functionality, and Docker image of each module are listed in Supplementary Table S2. Detailed description of each module is available in the Supplementary Methods (Supplementary File S1). To help users write their Methods section, a template is provided at https://github.com/hukai916/scATACpipe/blob/main/docs/template_of_method.docx including citations to all incorporated tools.

### 2.3 Case study

To demonstrate the functionality and reliability of our pipeline, we applied our pipeline to a public human scATAC-seq dataset with matched scRNA-seq data, and a plant scATAC-seq dataset without matched scRNA-seq data (Farmer et al., 2021). For brevity, here we only present the results from analyzing the human scATAC-seq data, while analysis results of the plant data are available as part of the online pipeline documentation (https://github.com/hukai916/scATACpipe#an-example-using-plant-genome-without-matched-scrna-seq-data).

#### 2.3.1 scRNA-seq data analysis

Five scRNA-seq datasets (Supplementary Table S3) of human peripheral blood mononuclear cells (PBMCs) generated by 10x Genomics were analyzed using the 10x Genomics Cell Ranger software (version 6.0.0, https://github.com/10Xgenomics/cellranger) and the Seurat (version 4.0.2) package (Hao et al., 2021). Briefly, using *cellranger count* with default settings, the scRNA-seq data was mapped to the human reference genome GRCh38 (Ensembl 98) (10x Genomics genome index, 2020-A released on July 7, 2020) and per-cell gene expression was quantified with the human GTF file (GENECODE release 32). A SoupChannel object for each sample was created from the 10x Genomics Cell Ranger output directory “outs” and ambient RNA contamination of each cell was determined and removed using SoupX (version 1.5.2) (Young and Behjati, 2020). A Seurat object was created by combining the ambient RNA-adjusted count matrices of the five samples. Subsequently, cells with fewer than 200 genes detected, cells with more than 12.5% of read counts from mitochondrial genes, and cells with fewer than 5% of read counts from ribosomal genes were excluded. Additionally, genes with detected expression in fewer than 0.1% of cells were removed. The Seurat object was then split into five Seurat objects by sample identities. Within each Seurat object, the ambient RNA-adjusted gene-by-cell matrix was processed using the Seurat package as follows. First, the matrix was log-normalized with the NormalizeData function and top 2,000 highly variable genes (HVGs) were identified using the FindVariableFeatures function. Then the expression of those HVGs was scaled by regressing out biases caused by cell-to-cell variations in the number of detected genes and percentage of read counts for mitochondrial genes using the ScaleData function. Dimension reduction was performed on those scaled expression data of those HVGs using the RunPCA function. UMAP embedding was performed with the top principal components as determined by the elbow method. Subsequently, doublets were determined and removed using the doubletFinder_v3 function from the DoubletFinder package (version 2.0.3) (McGinnis et al., 2019). Each of the doublets-removed gene-by-cell matrices was re-normalized and top 2000 HVGs were determined again for each Seurat object as above. Common HVGs across the five datasets were identified using the SelectIntegrationFeatures function and integration anchors were determined with the FindIntegrationAnchors functions using the Reciprocal PCA method. Next, the five datasets were integrated using the IntegrateData function. The integrated Seurat object underwent scaling, dimension reduction and UMAP embedding as above. A graph was constructed based on shared nearest neighbors of each cell in the integrated object using the FindNeighbors function. Cells represented by the nodes in the graph were clustered using the FindClusters function with a resolution of 0.7. Inference of cell types of individual cells were performed with bulk expression profiles of 29 purified human immune cell types (Monaco et al., 2019) as reference using the SingleR package (Aran et al., 2019). The cluster annotation was verified with known marker genes specific to each type of PBMCs (Zheng et al., 2017; Zhu et al., 2020; Cao et al., 2021; Liu et al., 2021; Waickman et al., 2021; Wang et al., 2021). The Seurat object was converted into a SummarizedExperiment object, which was used for integration with the human PBMC scATAC-seq data via scATACpipe. The Seurat object was slightly modified to meet the specific requirements of scATAC-pro for label transfer and MAESTRO for integration analysis. Note that tools for analyzing scRNA-seq data are not included in scATACpipe. Scripts for the scRNA-seq data analysis are available at GitHub (https://github.com/haibol2016/PBMC_scRNAseq_analysis).

#### 2.3.2 scATAC-seq data analysis

A human PBMC scATAC-seq dataset consisting of eight libraries generated by 10x Genomics (Supplementary Table S3) was analyzed using our scATACpipe. The pipeline was executed three times for comparisons, each using one of the three preprocessing sub-workflows and the same downstream analysis sub-workflow. Resource usage and time to run the pipeline for this case study can be found at GitHub (https://github.com/hukai916/scATACpipe#pipeline-info). Briefly, the fasta sequence file (Ensembl release 98) and GTF annotation file (GENCODE release 32) for the primary assembly of the human reference genome GRCh38 were manually downloaded from the Ensembl Genome Browser and GENCODE, respectively, to match those used for the scRNA-seq data analysis. The default module configuration (located under the directory, conf/modules. config) was modified by supplying the *marker_genes* parameter with a set of known marker genes of different types of human PBMCs (Zheng et al., 2017; Zhu et al., 2020; Cao et al., 2021; Liu et al., 2021; Waickman et al., 2021; Wang et al., 2021). An initial analysis of the scATAC-seq data was performed with paths to the reference genome sequence file and the GTF file being specified via Nextflow’s command-line parameters and the modified configuration file.

The HTML report from the initial analysis was examined to identify problematic libraries, low-quality cells, and artificial clusters. Specifically, the FastQC section was checked to identify libraries of poor sequencing quality; the Qualimap section was checked to identify libraries of poor alignment quality; the barcode correction section was checked to identify problematic libraries; the bivariate scattering plots and ridge plots were examined to determine the optimal cutoffs for cell filtering; the fragment size distribution per library was checked for poor-quality libraries; the UMAP plots showing doublet enrichment per library was checked to identify optimal doublet filtering parameters; the UMAP and tSNE plots were checked to optimize the parameter of clustering resolution; the heatmaps showing the cluster-sample confusion matrix and marker genes were checked to identify outlier libraries and artificial clusters. Consequently, the module configuration file was updated so that problematic libraries (PBMC_10K_C and PBMC_10K_X), and cells of low-quality (cells with unique nuclear fragment counts <3,000 or TSS enrichment scores <10) or forming artificial clusters were excluded from further analyses. The scATAC-seq data was re-analyzed with the updated configuration files by resuming the previous run. As such, a few rounds of exploratory downstream analyses were conducted with the module configuration file being updated according to the results of a previous run by resuming the latest run of the pipeline. A final clustering was performed with a resolution of 0.7. Unconstrained integration of the scATAC-seq data with the matched scRNA-seq data were performed with the SummarizedExperiment object for the scRNA-seq data being specified in the module configuration file. As a result of the unconstrained integration, the clusters of scATAC-seq data were preliminarily annotated with cell types. To perform the constrained integration, we added to the module configuration file the preliminary clustering information for T cells, NK cells, and that for other cell types for the scATAC-seq data. Constrained integration was performed with the updated configuration file by resuming the pipeline. As a result of the constrained integration, the cell types were updated for the clusters of scATAC-seq data with added gene expression information from the scRNA-seq data.

Without further modification of the module configuration file, the following analyses were subsequently performed by the pipeline. Pseudo-bulk replicates were generated based on the cluster assignments and a set of reproducible peaks was identified from the pseudo-bulk replicates. A PeakMatrix was then added to the ArchR project and marker peaks were identified for each cluster. Motif enrichment, motif deviation, and footprinting analyses were performed for cluster-specific marker peaks. Integrative analyses, including peak co-accessibility analysis, peaks-to-gene linkage analysis, and positive TF regulator analysis, were carried out to identify potential open chromatin interaction, potential *cis*-regulatory elements, and positive TF regulators in each cluster. The final pipeline configuration files used for the scATAC-seq data analysis are available at GitHub (https://github.com/hukai916/scATACpipe/tree/main#an-example-using-human-genome-with-matched-scrna-seq-data).

### 2.4 Comparison of scATACpipe with existing scATAC-seq data analysis pipelines

To demonstrate the unique merits of our scATACpipe, we compared its performance with that of two major pipelines for scATAC-seq data analysis, scATAC-pro (v1.5.0) (Yu et al., 2020) and MAESTRO (v1.5.1) (Wang et al., 2020). The same human PBMC scATAC-seq data mentioned in the case study was used for this purpose. Whenever possible, the same software tools and parameters were applied to the three comparative analyses that were executed on the same high-performance computing clusters. Parameter settings and analysis scripts for running scATAC-pro and MAESTO are available in Supplementary File S2, S3, respectively. The metrics we considered were ease and flexibility of parameter configuration, usage of computing resources, completeness of functionalities, and biological relevance of final analysis outcomes.

We carried out all but footprinting analysis of the scATAC-seq data with the debugged and modified scATAC-pro pipeline. The reasons that we had to modify the scripts are as follows: 1) parameters for setting memory and threads are hardcoded with improper defaults in multiple modules; 2) the downstream modules for differential accessibility analysis, GO term enrichement analysis, and footprinting analysis failed due to intrinsic errors.

MAESTRO (Wang et al., 2020) was applied to analyzing both the human PBMC scATAC-seq data and the matched scRNA-seq data, and integrating them together. Neverthless, MAESTRO cannot properly handle multi-sample experiments with batch effects for either scRNA-seq or scATAC-seq data. To facilitate comparisons of the results generated from different pipelines, we modified the Seurat object from the analysis of scRNA-seq data for scATACpipe integration analysis (see Section 2.3.1) in accordance with the MAESTRO requirements for integration.

## 3 Results

### 3.1 Identification and characterization of cell type-specific open chromatin landscape in human PBMCs using the scATACpipe

To facilitate comprehensive and reproducible analysis of the most popular 10x Genomics scATAC data, we developed a Nextflow pipeline, scATACpipe (Figure 1). As a demonstration, we analyzed one human PBMC scATAC-seq dataset from eight libraries generated by 10x Genomics, using our scATApipe. We validated all preprocessing sub-workflows and the common downstream analysis sub-workflow. The three different preprocessing sub-workflows produced largely similar fragment files (Supplementary Figure S1) and cell barcodes (Supplementary Figure S2), from which the common downstream analysis sub-workflow derived consistent cell clusters (Supplementary Figure S3). For simplicity, we mainly showed results from the default preprocessing and its downstream analysis in the main text, unless otherwise stated.

For any high-throughput sequencing data, library-level QC is of general importance for assessing the overall sequencing quality and library quality. Quality checking of the raw sequencing data showed that all scATAC-seq data from the eight libraries had high sequencing quality, although two of the libraries constructed from only 500 and 1,000 nuclei had high percentages of over-represented sequences. Additional support for high quality of the libraries are that adaptor content was low (∼2%) in all libraries and only a small percentage of barcodes needed correcting.

**FIGURE 2:**
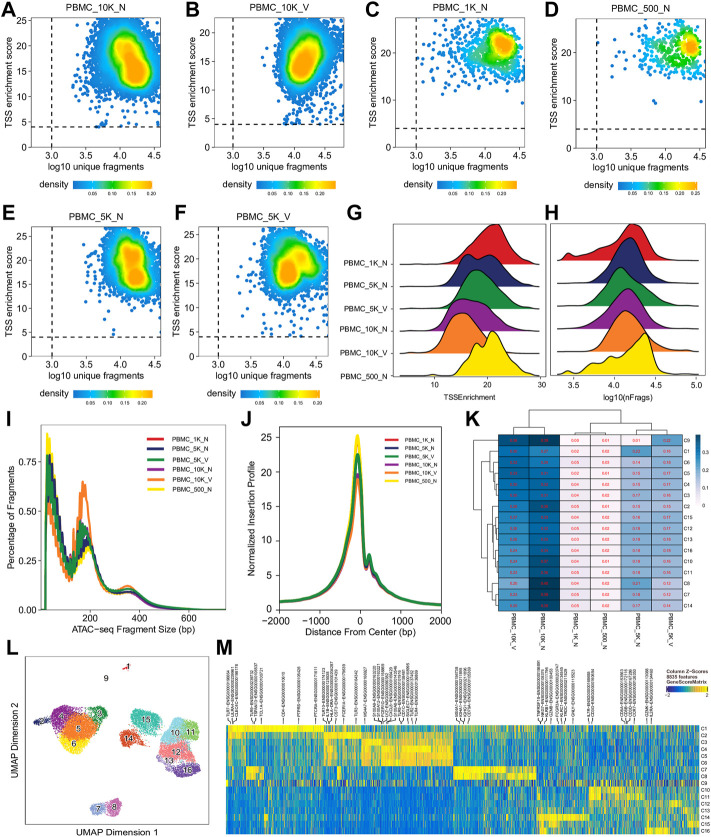
Cell- and library-level QC of the human PBMC scATAC-seq data. Fragment files were generated for the six samples (outliers excluded) by the default preprocessing sub-workflow, and analyzed by the common ArchR-based downstream analysis sub-workflow. **(A–F)** Scatter plots showing bivariate distributions of TSS enrichment scores and log_10_ (unique fragments) of individual cells in each of the six libraries. **(G,H)** Ridge plots showing distributions of TSS enrichment scores and log_10_ (unique fragments) of individual cells per library, respectively. **(I)** Density plots showing insert size distributions per library. **(J)** Normalized insertion profiles along ±2-kb regions flanking TSSs. **(K)** Clustered heatmap showing proportions of cells per cluster from each library, with each row summing up to 1. **(L)** UMAP plot showing 16 clusters identified from the scATAC-seq data. **(M)** Heatmap showing cluster-specific marker genes across 16 clusters. Noticeably, cluster C9 has a noisy, weak pattern of marker genes, which suggests that it is very likely formed by doublets.

For single cell sequencing data, QC at the single-cell level is essential to exclude low-quality cells for further analyses. Single cell QC showed that almost all cells in different libraries were of high quality, i.e., TSS enrichment score >4 and unique fragment count > 10^3^, although all had a wide range of unique fragment counts (10^3^–10^5^) (Figures 2A–F,H, Supplementary Figure S1, S4) and a relative broad distribution of TSS enrichment scores (Figures 2A–G, Supplementary Figure S1S4). Relationship between TSS enrichment scores and the number of unique fragments in individual cells for each library are shown in Figures 2A–F, Supplementary Figure S1S4. All libraries had similar distributions of insert sizes with expected laddering, periodic patterns (Figure 2I, Supplementary Figure S1S4) and insertion profiles (Figure 2J) around TSSs, indicating they were of high quality ATAC-seq libraries. However, after an initial analysis using the scATACpipe with the 10x Genomics Cell Ranger ATAC-based preprocessing sub-workflow, we observed that cells in 11 of the 25 clusters were predominantly from two (PBMC_10K_C and PBMC_10K_X) of the eight libraries, while cells in the other 14 clusters had similar representation across all the eight libraries (Supplementary Figure S5). These results suggest that those two libraries were different from the rest and the batch effects could not be completely corrected by Harmony (Zheng et al., 2017). Therefore, those two outlier libraries were excluded from analyses by the other two preprocessing sub-workflows and all downstream analyses.

**FIGURE 3:**
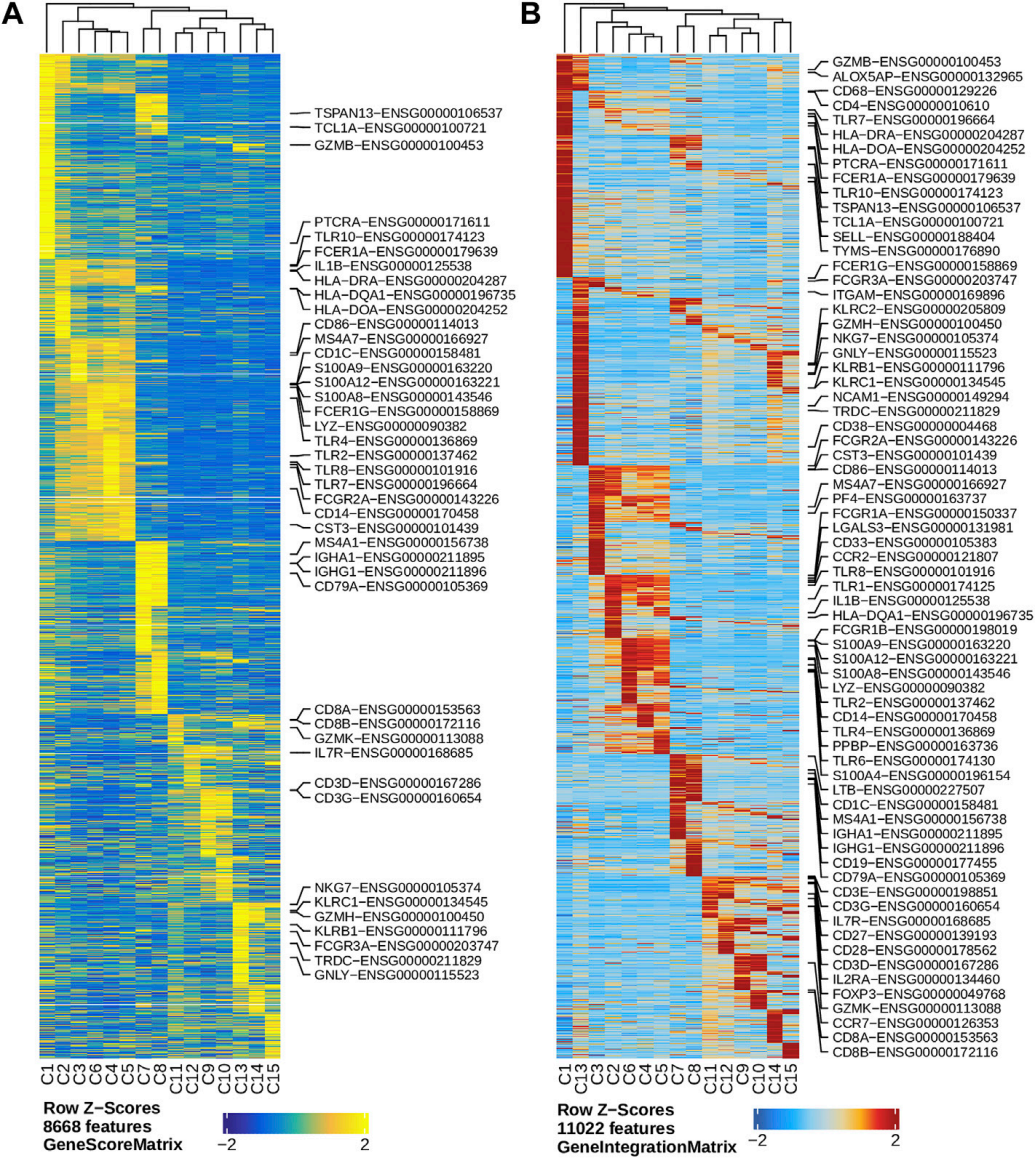
Marker genes across clusters identified in the human PBMC scATAC-seq data. **(A)** Heatmap showing standardized gene scores (Z-scores) of 8,668 marker genes across the 15 clusters. **(B)** Heatmap showing standardized pseudo expression (Z-scores) of marker genes across the 15 clusters. Known marker genes in the form of gene symbol-Ensembl ID are labelled on the right of each heatmap.

We performed dimension reduction, batch correction, and clustering analyses of data from the remaining six libraries, and identified similar numbers of clusters and cluster-specific marker genes using the three different preprocessing sub-workflows. Specifically, we identified 18 (Supplementary Figure S5), 17 (data not shown), and 16 (Figure 2L) clusters from fragment files generated by the 10x Genomics Cell Ranger ATAC software-based preprocessing sub-workflow, the Chromap-based preprocessing sub-workflow, and the default preprocessing sub-workflow, respectively. Supplementary Figure S5 shows that all clusters except C10 and C15, derived from fragment files via 10x Genomics ATAC software-based preprocessing sub-workflow, were well represented by cells from each of the six libraries. Similarly, we found one and two clusters, not well represented by cells from each library, among clusters derived from the default preprocessing sub-workflow and the Chromap-based preprocessing sub-workflow, respectively. Those clusters not well represented by cells from every individual library were most likely technical artefacts, possibly formed by unremoved doublets. This observation is further supported by cluster-specific marker gene analysis, which revealed atypical clusters with noisy and weak patterns of marker genes in a gene heatmap (e.g. cluster C9 in Figure 2M). After excluding those atypical clusters, we identified 15 highly reproducible clusters by each of three preprocessing sub-workflows. The marker gene analysis based on the GeneScoreMatrix identified 8,668 cluster-specific marker genes, including many known cell type-specific marker genes, such as CD79A, CD14, and CD8A (Figure 3A).

**FIGURE 4:**
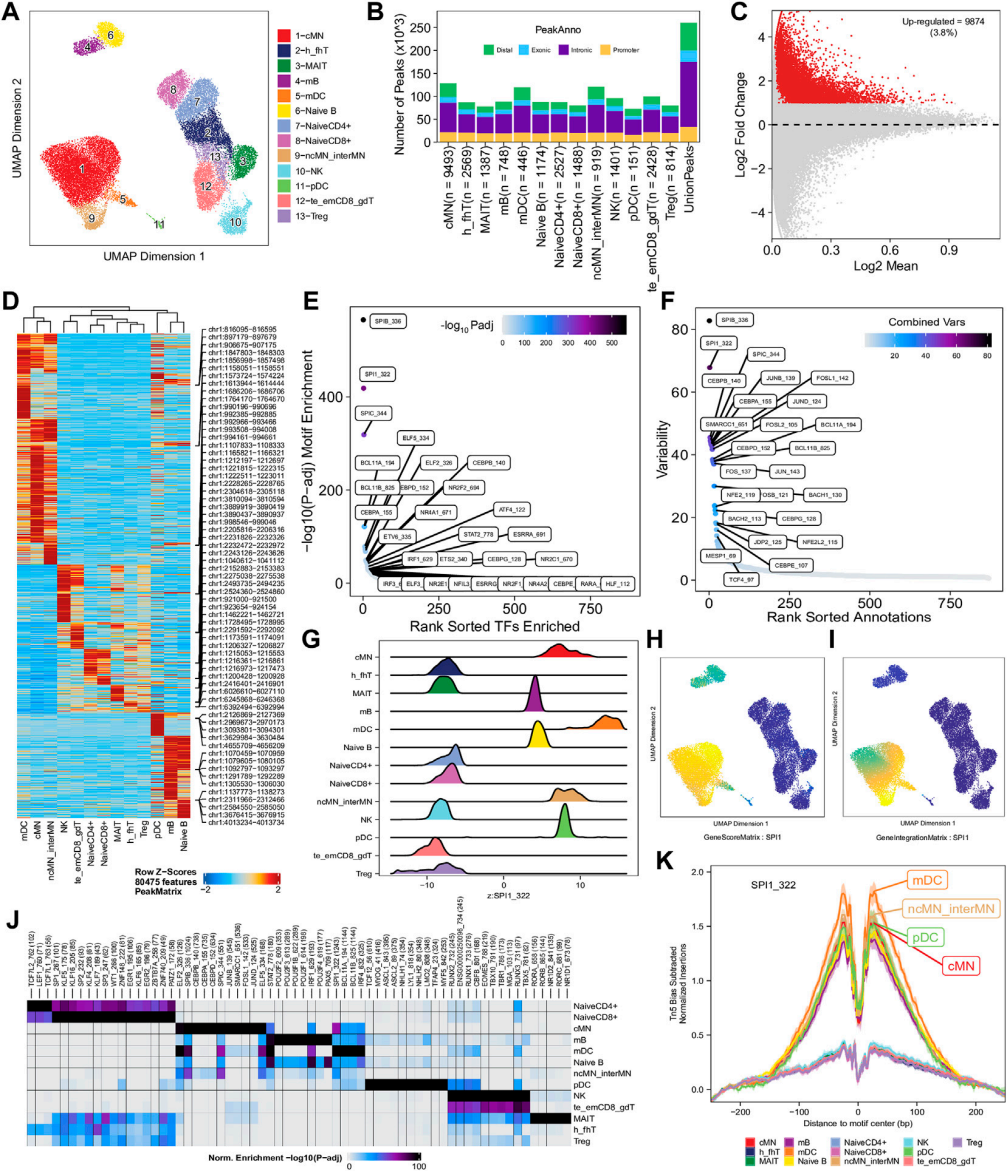
Functional analysis of annotated clusters of the human PBMCs. **(A)** UMAP plot showing annotated clusters identified in the human PBMCs by integrating with the human PBMC scRNA-seq data. cMN, classic monocytes; h_fhT, T helper and T follicular helper cells; MAIT, mucosal-associated invariant T cells; mB, memory B cells, mDC, myeloid dendrtic cells; Naive B, naive B cells; NaiveCD4+, naive CD4^+^ T cells; NaiveCD8+, naïve CD8^+^ T cells; ncMN_interMN, non-classic monocytes and intermediate monocytes; NK, natural killer cells; pDC, plasmacytoid dendritic cells; te_emCD8_gdT, terminal effector CD8^+^ T cells, effector memory CD8^+^ T cells and γδT cells; Treg, T regulatory cells. **(B)** Distribution of reproducible peaks among different genomic features (promoter, intronic, exonic, and distal regions) in each cell type. **(C)** MA plot showing peaks preferentially accessible in intermediate and non-classic monocytes (FDR <0.01 and log_2_FC ≥ 1). **(D)** Heatmap showing 80,475 marker peaks across the 13 annotated clusters. **(E)** Dot plot showing top motifs enriched among marker peaks of intermediate and non-classic monocytes (ncMN_interMN). **(F)** Dot plot showing motifs of top variability scores across all the 13 cell types determined by ChromVAR. **(G)** Ridge plots showing distributions of the Z-score of motif deviation scores for SPI1_322 in each cell type. **(H,I)** UMAP plots showing gene scores and pseudo expression of a monocyte-specific TF, SPI1, whose motif is highly enriched in monocytes (cMN, interMN, and ncMN) and dendritic cells (mDC and pDC) and is of high deviations across clusters. **(J)** Heatmap showing normalized enrichment score, -log_10_ (adjusted *p*-value), of top TF motifs across the different cell types. **(K)** Aggregate footprints of SPI1_322 in each cell type.

To enhance the analysis of the scATAC-seq data, we performed an integrated analysis with matched PBMC scRNA-seq data. Cell clusters identified from scRNA-seq data were annotated by using a correlation-based method (see Section 2.3.1) (Supplementary Figure S6). After the initial unconstrained integration and the subsequent constrained integration, 11,022 marker genes from the GeneIntegrationMatrix (Figure 3B) were identified in the 15 clusters. As expected, integration with the scRNA-seq data resulted in more marker genes identified from the scATAC-seq data. The annotation labels of scATAC-seq cell clusters were deduced from those of the matched scRNA-seq cell clusters (Figure 4A), resulting in 13 annotated cell clusters (Figure 4A). With the multiple pseudo-bulk replicates for each cell cluster, 260,168 reproducible peaks were identified. The distribution of reproducible peaks across different genomic features is shown in Figure 4B. Figure 4C displays a MA plot showing 9,874 peaks preferentially detected in intermediate and non-classic monocytes, while Figure 4D displays 80,475 marker peaks across the 13 annotated cell clusters.

With those 80,475 marker peaks, we identified potential transcription factors playing roles in each cell types by motif enrichment, motif deviation, and footprinting analyses. Top enriched motifs among marker peaks of intermediate and non-classic monocytes are shown in Figure 4E, while top enriched motifs across cluster-specific marker peaks are shown in Figure 4J. Motifs of large ChromVAR deviation scores across all marker peaks are shown in Figure 4F. SPI1 (also known as PU.1), a key TF controlling differentiation of myeloid and lymphoid cells (Scott et al., 1994; DeKoter and Singh, 2000), is one of the cell type-preferred transcription factors identified here. It is highly expressed in monocytes and dendritic cells, lowly expressed in mature B cells, but not expressed in T cells or NK cells (Lloberas et al., 1999; DeKoter and Singh, 2000) (https://www.proteinatlas.org/ENSG00000066336-SPI1). Consistent with the literature, we successfully identified SPI1_322 as one of the top enriched motifs in monocytes, dendritic cells, and B cells (Figure 4E and data not shown), and one of the motifs with top variability in terms of ChromVAR deviation scores (Figure 4F). Additionally, the standardized ChromVAR deviation scores (Z-scores) are very high in dendritic cells and monocytes, slightly high in naïve and memory B cells, but extremely low in T cells and NK cells (Figure 4G). Consistently, plots showing gene activity scores (Figure 4H), pseudo gene expression (Figure 4I), and aggregated footprints of SPI1 (Figure 4K) also indicate that SPI1 is highly expressed in dendritic cells and monocytes, lowly expressed in B cells, but not expressed in T cells or NK cells.

**FIGURE 5:**
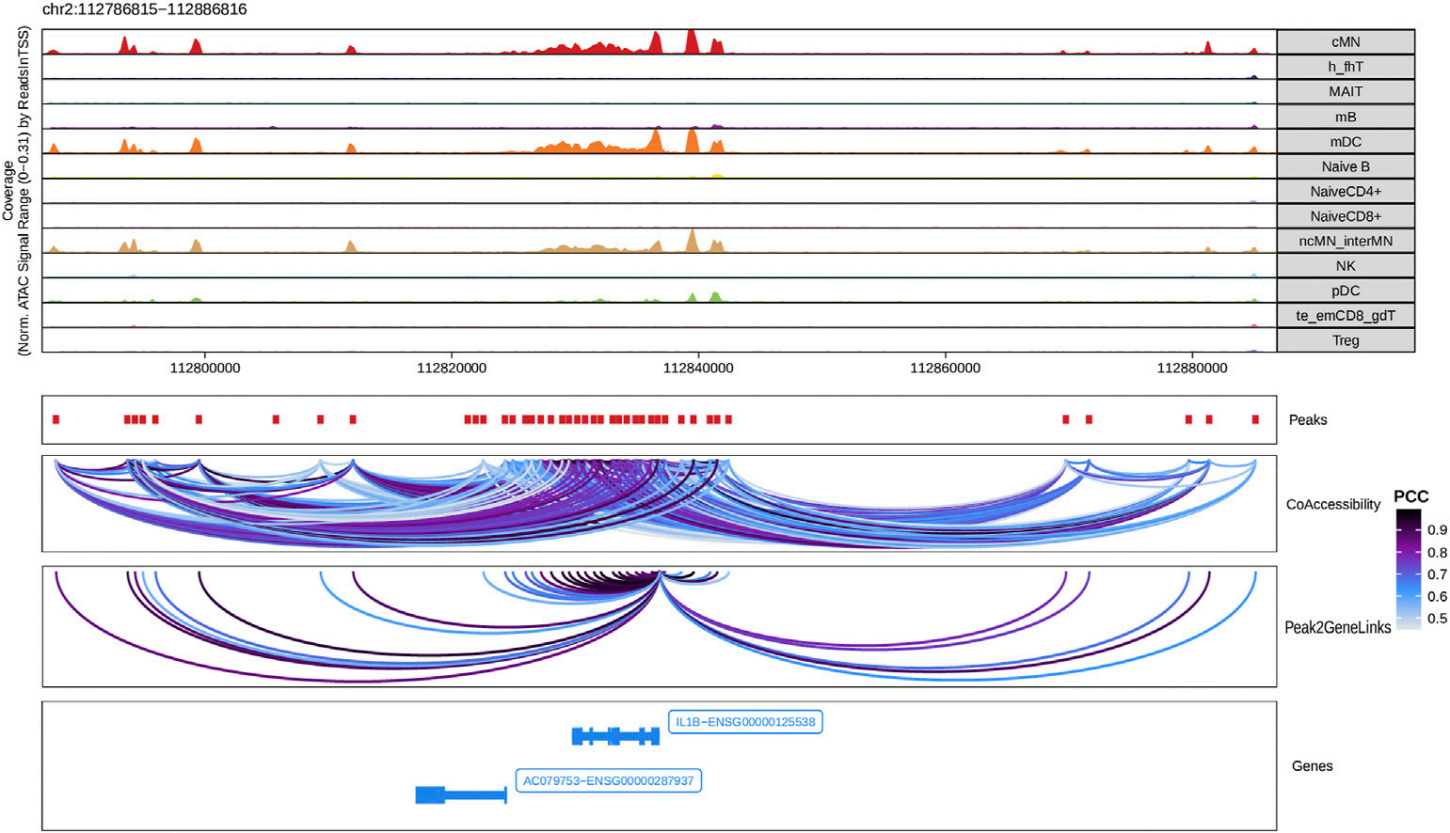
Co-accessibility and peak-to-gene links in a *IL1B*-containing genomic region. The top panel shows the normalized read coverage in the genomic region of each cell type. The second panel shows peaks represented by red boxes. The third panel shows peak pairs, linked by colored arches, with co-accessibility scores (Pearson correlation coefficients) greater than 0.5. The fourth panel shows peaks linked to the *IL1B* promoter peak with co-accessibility score greater than 0.5. The fifth panel shows gene models of *IL1B* and AC079753 in this region.

**FIGURE 6:**
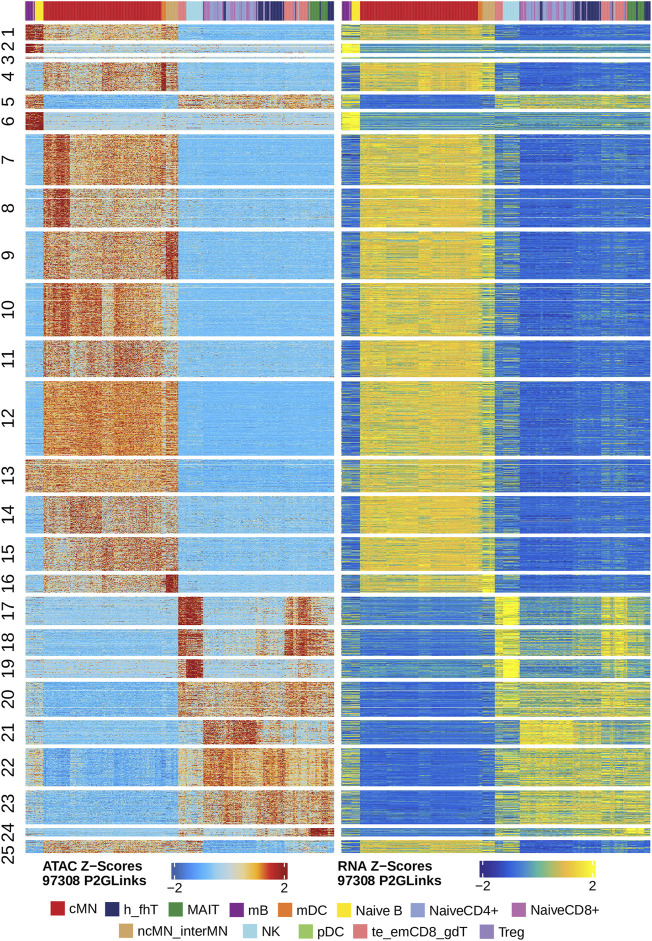
Gene scores and pseudo expression of genes linked with peaks are highly consistent in different types of the human PBMCs. Heatmaps on the left and right panels show gene scores (derived from the scATAC-seq data) and pseudo expression (transferred from the scRNA-seq data) of genes linked to the peaks, respectively. Color bars on the top of each heatmap represent the PBMC clusters derived from the scRNA-seq data.

**FIGURE 7:**
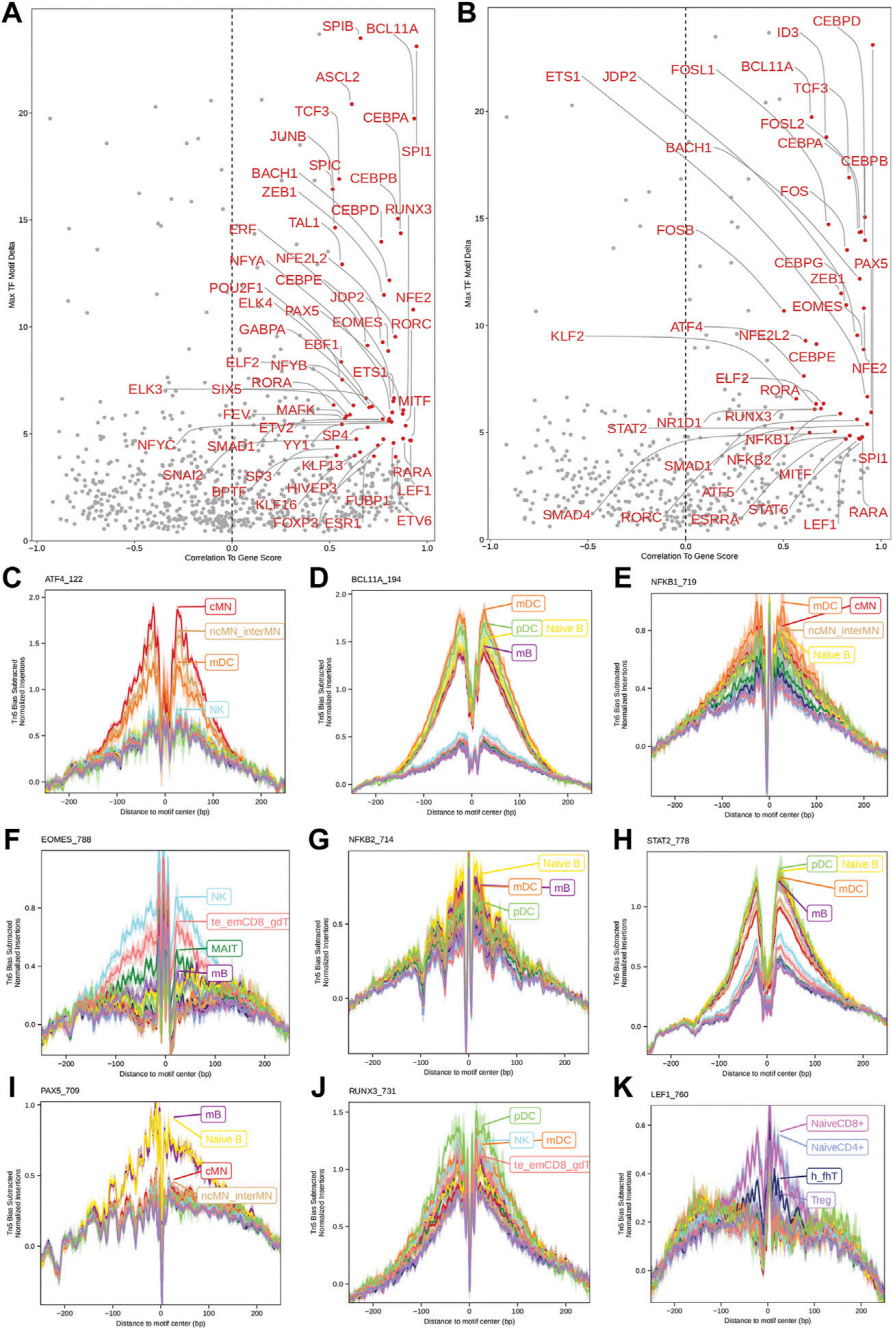
Identification of positive TF regulators in PBMCs based on gene scores or pseudo gene expression. **(A,B)** Positive TF regulators with red labels were identified by correlating gene scores and pseudo gene expression of TFs with deviation scores of their motifs across all human PBMCs, respectively. Seventeen positive TF regulators were identified by both methods. **(C–K)** Aggregate footprints of nine positive TF regulators preferentially functioning in different cell types.

Next, we identified genome-wide potential chromatin interactions and potential gene expression regulation mechanisms by performing a series of integrative analyses, including co-accessibility analysis, peak-to-gene linkage analysis, and positive TF regulator analysis. Here we use the *IL1B* gene as an example. IL1B is a key pro-inflammatory cytokine, mainly expressed in monocytes and mDCs among human PBMCs (https://www.proteinatlas.org/ENSG00000125538-IL1B) (Gardella et al., 2000; Hadadi et al., 2016). It triggers monocyte activation, inducing cytokine release and differentiation into macrophages and dendritic cells (Schenk et al., 2014). Furthermore, in monocytes, *IL1B* is a direct target of SPI1, which constitutively binds to two distinct sites (−50 to −39 and −115 to −97) upstream of the TSS of *IL1B* (Kominato et al., 1995; Adamik et al., 2013). Shown in Figure 5 are normalized coverage for each cell type, peaks across all cell types, peak co-accessibility, and peaks-to-gene (*IL1B*) links in a 100-kb genomic region, centering on the TSS of the *IL1B* gene. Those peaks linked with *IL1B* are potentially involved in regulating *IL1B* expression (Shirakawa et al., 1993; Kominato et al., 1995; Adamik et al., 2013). In line with that the model used by ArchR to infer gene activity is accurate (Granja et al., 2021), we found gene scores and pseudo expression of genes associated with 97,308 peak-to-gene links were highly consistent (Figure 6). It is also worth mentioning that we identified 53 and 36 positive TF regulators by positive TF regulator analysis based on gene scores and pseudo expression of TFs, respectively, with 17 positive TF regulators in common (Figures 7A,B). Among the common positive TF regulators were ATF4, BCL11A, NFKB1, NFKB2, EOMES, STAT2, PAX5, RUNX3, LEF1, and SPI1. The cell types where these positive TF regulators play their regulatory roles were corroborated by footprinting analysis. Figures 7C–K shows footprints of nine positive TF regulators that are preferentially active in different cell types. The results of our positive TF regulator analysis are consistent with previous publications (Gupta et al., 1999; Park et al., 2000; Hayden et al., 2006; Cobaleda et al., 2007; Medvedovic et al., 2011; Yu et al., 2012; Steinke and Xue, 2014; De Silva et al., 2016; Boto et al., 2018; Dorrington and Fraser, 2019; Shimizu et al., 2019; Mukherjee et al., 2020; Qiu et al., 2020). Here we just take PAX5 as an example. In peripheral blood, PAX5 is only expressed in B cells (https://www.proteinatlas.org/ENSG00000196092-PAX5) (Fuxa and Busslinger, 2007), as the guardian of B cell identity and function (Cobaleda et al., 2007; Medvedovic et al., 2011). In consistency with the literature, we identified PAX5 as a B cell-specific transcription factor by positive TF regulator analysis (Figures 7A,B) and footprinting analysis (Figure 7I).

### 3.2 Comparison of scATACpipe with other pipelines

We carried out all but footprinting analysis of the scATAC-seq data with the debugged and modified scATAC-pro pipeline, and all analyses implemented in MAESTRO. In summary, both scATAC-pro and MAESTRO successfully preprocessed data of all six samples, and performed sample-level and cell-level QC (Supplementary Figure S7, S8). scATAC-pro properly integrated the individual samples (Supplementary Figure S7), but MAESTRO failed to do so because it lacks the functionality to remove the batch effects (Supplementary Figure S8). As a result, the major cell types in the human PBMCs were tightly clustered into 19 groups by scATAC-pro, though some potential doublets (some cells in clusters 10, 15, 16, 17 and 18) were carried over to the final analysis (Supplementary Figure S7). Annotation of most cell types were further supported by GO term enrichment analyses of genes preferentially expressed in each cluster (Supplementary Figure S7 and data not shown). However, the same PBMCs were clustered in 42 groups by MAESTRO, with the cells of the same types forming smaller clusters far from each other in the UMAP plots (Supplementary Figure S8). Both scATAC-pro and MAESTRO identified candidate TFs playing roles in each cell cluster using different methods (Supplementary Figure S7, S8). The candidate TFs identified in each cell type by the three pipelines were largely similar. Like scATACpipe, scATAC-pro predicted co-accessibility between regional open chromatin regions (Supplementary Figure S7), but MAESTRO lacks this functioinality. Apparently, the co-accessibility between open chromatin regions predicted by scATAC-pro looks very different from that predicted by our scATACpipe for the genomic region containing the *IL1B* gene (Supplementary Figure S7 and Figure 5).

It is worth mentioning that the three pipelines called different numbers of cells in each sample (Supplementary Table S4). scATACpipe and the Cell Ranger ATAC software report similar cell numbers, while MAESTRO reports systematically higher cell numbers than 10x Genomics Cell Ranger ATAC software with default parameters by 10–20%. Surprisingly, scATAC-pro called 57.2–189% more cells than 10x Genomics Cell Ranger ATAC software even though it uses the Cell Ranger cell calling algorithm re-implemented in scATAC-pro. Additionally, these pipelines detected very different numbers of consensus peaks. scATAC-pro detected only 123,909 consensus peaks, while our scATACpipe and MAESTRO detected 260,168 and 388,730 consensus peaks, respectively.

Besides comparing the biological relevance of the final output of the three pipelines, we also considered other important metrics: ease and flexibility of parameter configuration, completeness of functionalities, and usage of computing resources. Here, we focus on comparing their usage of computing resources while leaving the rest to the Discussion section. A summary of computing resources and runtime of the scATAC-pro and MAESTRO is shown in Supplementary Table S4, while that of our scATACpipe is available as online documentation (https://github.com/hukai916/scATACpipe#pipeline-info). In short, MAESTRO required the largest memory for its SingleQCMappability step when processing sample PBMC_10K_N (347 GB) whereas scATAC-pro used the longest CPU time for preprocessing sample PBMC_10K_V (225 h). As for runtime, scATACpipe and MAESTRO with the chrommap option are faster than other settings, and scATAC-pro is the slowest.

## 4 Discussion

ScATAC-seq has become one of the most widely used methods for deciphering the role of chromatin accessibility in regulating gene expression at the single-cell level. Data generated by scATAC-seq is extremely sparse, noisy, and high-dimensional, which poses analytic challenges (Chen et al., 2019; Fang et al., 2021). To overcome these challenges, we have built a scalable, portable, and comprehensive pipeline, scATACpipe, using the Nextflow workflow management system. Our pipeline provides three options for preprocessing 10x Genomics scATAC-seq data from raw fastq files to filtered fragment files. Depending on users’ preference, raw scATAC-seq data can be processed using the carefully tailored default sub-workflow which integrates the current best analytic practices, the commercially supported 10x Genomics Cell Ranger ATAC software-based sub-workflow, or the recently developed Chromap-based sub-workflow (Zhang H. et al., 2021). The fragment files generated by the three sub-workflows are largely similar, with slight differences due to the adoption of different tools and parameters (Zhang H. et al., 2021) (Supplementary Figure S1, S2). The default preprocessing option allows users to configure the largest number of parameters, while the Cell Ranger ATAC-based sub-workflow provides the least control over parameter settings. In light of that the Chromap-based sub-workflow is the most time-efficient (Zhang H. et al., 2021), we implemented functionalities in the default sub-workflow to enable users to split large fastq files into smaller chunks to speed up preprocessing. All three sub-workflows support adapter trimming, read alignment, alignment deduplication, barcode correction, cell calling, and fragment file generation.

Compared to preprocessing, the downstream analysis of scATAC-seq data is more data-driven, requiring more step-specific inputs from users. Our pipeline implemented an ArchR-based sub-workflow for downstream analysis with each major function as an individual module. This sub-workflow not only generates gene annotation and genome annotation objects for species that ArchR does not internally support, but also creates Arrow files, identifies and removes doublets/multiplets, creates ArchR project, and performs cell QC and filtering. Moreover, it also provides a wide variety of core analysis modules, such as dimension reduction, batch correction, clustering and embedding, optional integration with matched scRNA-seq data, marker gene identification, peak calling, marker peak identification, differential peak analysis, peak-set based analyses (motif enrichment analysis, motif deviation analysis and footprinting analysis), co-accessibility analysis, peak-to-gene linkage analysis for gene activity inference, positive TF regulator inference, and potential cell trajectory inference. Nevertheless, due to the uniqueness of each dataset, the default configuration of our scATACpipe for the downstream analysis is meant for users to get some preliminary results. Following an initial run, users are advised to modify the configuration file by tuning relevant parameters meticulously and resume the downstream analysis, which might need to be performed iteratively to achieve optimal results. It is worth mentioning that we added quite a few functionalities to enhance the usage of the ArchR package. We implemented a few R functions to streamline the process of generating and installing BSgenome packages for any sequenced genome assemblies and preparing gene annotation and genome annotation objects for any annotated genome assemblies. These functions make it easier for users to apply ArchR to any eukaryotic species with annotated genomes. Compared to the original ArchR, our downstream sub-workflow also provides additional flexibility for doublet identification and cell filtering. Particularly, besides the ArchR’s built-in functionality for doublet removal, it utilizes the AMULET package to determine multiplets based on read count per genomic locus (Thibodeau et al., 2021). Additionally, our scATACpipe allows users to exclude cells in certain clusters determined by preliminary analyses, along with low-quality cells.

Also importantly, while Cell Ranger ATAC software-based sub-workflow outputs an interactive HTML report, our pipeline generates a comprehensive, interactive HTML report for both the default preprocessing sub-workflow and the downstream analysis sub-workflow. This comprehensive report includes sections on QC for raw sequencing data, adaptor trimming, barcode correction, read alignment, alignment deduplication, valid cell filtering, and each major ArchR analysis step. Furthermore, our pipeline generates cluster-specific BED, BAM, and/or BigWig files for visualization in genome browsers, in addition to generating track views for specific genomic regions of interest by using ArchR functions.

In terms of ease and flexibility of parameter configuration, our scATACpipe is the best, followed by MAESTRO, and then scATAC-pro. Our scATACpipe allows users to configure nearly every possible parameter, including those for specifying computing resources, by setting command-line parameters, and by editing configuration files including workflow-level configuration file (nextflow.config) as well as module-level configuration files (conf/base.config and conf/modules.config). In addition, scATACpipe provides an intutitive web application for setting major parameters and generating a configuration file. In contrast, MAESTRO produces a configuration file for each sub-workflow via running a corresponding initiation command, where a set of command-line parameters can be set. The resulting configuration file can be further edited before executing the sub-workflow. However, many other MAESTRO parameters are not configurable. Especially, MAESTRO offers neither parameters for setting default resource usage of individual tasks nor a retry mechasim that automatically requests for more resources once the last limits are reached. As for scATAC-pro, although it is flexible in terms of software selection for each step, it only allows a very limited set of parameters to be configured mainly via editing a configuration file (configure_user.txt). Some important parameters, including those for specifying memory and threads, are hard-coded and thus are not configurable unless users modify the module scripts. In addition, it is not managed by any workflow management engine. Thus, it is not robust and cannot resume an analysis from where errors occur.

Our scATACpipe is the most functionality-rich and optimal workflow among all existing tools and workflows for scATAC-seq data analysis (Supplementary Table S1), followed by scATA-pro (Yu et al., 2020) and then MAESTRO (Wang et al., 2020). Noticeably, the latter two pipelines can only support human and mouse scATAC-seq data analysis. The three pipelines share a majority of functionalities for preprocessing. However, unlike scATACpipe and MAESTRO, scATAC-pro does not have a module for cell barcode correction. One common weakness of scATAC-pro and MAESTRO is that they do not make full use of parallel computing. They merge fastq files from different lanes/runs for the same library at the beginning, which makes subsequent processing time-consuming and more memory-demanding, especially during BAM file sorting. As a consequence, they cannot efficiently handle large scATAC-seq data. Our scATACpipe and scATAC-pro, unlike MAESTRO, does not have a sub-workflow for analyzing scRNA-seq data, output of which can be integrated with scATAC-seq data to facilitate the analysis of the latter. However, the scRNA-seq sub-workflow of MAESTRO is not well implemented since it cannot correctly handle multi-sample experiments with batch effects (Supplementary Figure S9). All the three pipelines have functionalities for peak calling, generating count matrices, integrating multi-sample scATAC-seq data, differential accessibility analysis, and integrating scRNA-seq data with scATAC-seq data. The underlying algorithms used by scATAC-pro and MAESTRO are similar and not optimal, which are very different from those of scATACpipe (Granja et al., 2021). Specifically, both scATAC-pro and MAESTRO perform initial peak calling and generate a count matrix using aggregated data from cells in each sample separately and then merge those individual peak files to get consensus peaks and reconstruct a count matrix for clustering. The sparsity and noisiness of scATAC-seq data make peak calling based on individual samples, especially those with lower sequencing depth and cell numbers, less robust and sensitive (Fang et al., 2021; Granja et al., 2021). In contrast, scATACpipe divides a genome into non-overlapping 500-bp bins and generates a bin-by-cell count matrix for dimension reduction and clustering (Granja et al., 2021), followed by peak identification for each cluster and consensus peak generation. Consequently, scATACpipe generates the most accurate results of downstream analysis. Neither scATAC-pro nor MAESTRO fully leverages the matched scRNA-seq data. Instead, they only use the transferred scRNA-seq labels and/or expression data for the visualization of cell clusters. On the contrary, scATACpipe utilizes integrated scRNA-seq data for more comprehensive analysis such as cluster annotation, peak2GeneLinkage analysis, positive TF regulator analysis, and trajectory inference. Furthermore, it is not appropriate that both scATAC-pro and MAESTRO directly apply Seurat’s algorithms for scRNA-seq data analysis to differential accessibility analysis, given that scATAC-seq data is essentially binary in nature. When it comes to other downstream analyses, MAESTRO cannot perform doublet removal, batch effect correction, footprinting analysis, ChromVar-based motif deviation analysis, co-accessibility analysis, or trajectory inference, which are all offered by scATACpipe. On the other hand, scATAC-pro does not provide trajectory inference, although it has exclusive GO term enrichment analysis. Its modules for differential accessibility analysis and footprinting analysis are currently not functional and its downstream analysis sub-pipeline cannot be directly applied to analyzing integrated data of multiple samples.

In conclusion, scATACpipe is an open-source Nextflow-based pipeline for performing end-to-end analysis of large-scale scATAC-seq data. It enables users to conduct flexible preprocessing, all-level QC, and comprehensive downstream analysis of 10x Genomics scATAC-seq data for different species across various computing environments. With all functionalities implemented in one pipeline, it eliminates the need to use multiple tools to perform step-by-step analysis, which is both time consuming and error prone. In this work, we illustrated the utility, flexibility, versatility, and reliability of our pipeline, and demonstrated that our scATACpipe outperforms two other workflows in terms of configurability, scalability, accuracy, and streamlined downstream analysis. We foresee that it will benefit many researchers seeking to understand how chromatin accessibility relates to cellular heterogeneity.

## Data Availability

Publicly available datasets were analyzed in this study. This data can be found here: Supplementary Table S3.
