# Supplementary material for: scATACpipe: A nextflow pipeline for comprehensive and reproducible analyses of single cell ATAC-seq data
Source: Front Cell Dev Biol. 2022 Sep 27;10:981859. doi: 10.3389/fcell.2022.981859 (PMC9551270; doi:10.3389/fcell.2022.981859)
Supplement: Supplementary file 1 [file DataSheet1.ZIP › supplementary/File S1.Supplementary methods.docx]

# scATACpipe: A Nextflow Pipeline for Comprehensive and Reproducible Analyses of Single Cell ATAC-seq data

Kai Hu^1,†^, Haibo Liu^1,†^, Nathan Lawson^1^, Lihua Julie Zhu^1,2,*^

^1^ Department of Molecular, Cell and Cancer Biology, University of Massachusetts Chan Medical School, 364 Plantation Street, Worcester, MA 01605, USA

^2^ Program in Molecular Medicine, Program in Bioinformatics and Integrative Biology, University of Massachusetts Chan Medical School, Worcester, MA 01605, USA

^†^ These authors have contributed equally to this work.

*** Correspondence:**

Lihua Julie Zhu

Julie.Zhu@umassmed.edu

**Supplementary Methods**

## Modules for preprocessing

## Default customized data preprocessing modules

#### Preparing reference genome index

Given the path to a reference genome sequence file in fasta format as a command-line parameter, modules PREP_GENOME and BWA_INDEX are invoked to remove scaffolds for alternative haplotypes, if any, from the genome assembly and prefix the chromosome/scaffold names with “chr” if not present, and build the genome index from the resulting primary assembly. The reason to remove scaffolds for alternative haplotypes is to reduce unnecessary multi-mapping. Alternatively, users can specify the name of the genome assembly or species as a command-line option to automatically download the reference genome sequence from the UCSC (Karolchik et al., 2009) or ENSEMBL (Newman et al., 2018) Genome Browser with the DOWNLOAD_FROM_UCSC or DOWNLOAD_FROM_ENSEMBL modules.

This step is skipped if users specify a pre-built genome index as a command-line parameter. In this case, users must ensure that the genome index was built from a primary assembly of a reference genome of interest and is compatible with the aligner to be used. Of note, the default preprocessing sub-workflow uses BWA (Li and Durbin, 2009) for read alignment.

#### Preparing fastq files

Demultiplexed raw 10x Genomics scATAC-seq data for a library is usually composed of four fastq files per sample, with two for paired-end reads (R1 and R3) of genomic DNA, one for cell barcodes (R2) identifying cell origin of matching genomic DNA reads, and one for sample indices (I). Currently, scATACpipe only supports paired-end scATAC-seq data analysis because the library insert size can be easily inferred from paired-end sequencing data, which is a commonly used metric of ATAC-seq data quality (Buenrostro et al., 2013;Ou et al., 2018). To run this module, users provide a CVS file with each row containing four columns, i.e., the absolute paths to R1, R2, and R3 from each individual run of each sample, and a unique sample name. If multiple lane/run sequencing data is provided for a sample, the corresponding fastq files for each lane/run must be supplied in separate rows with the same sample name. Importantly, if users have sequencing data from multiple libraries on the same sample, the sample name for each library should be distinct to avoid collapsing data from different libraries. The INPUT_CHECK_FASTQ sub-workflow checks if the input CSV file is valid. To speed up the preprocessing of large fastq files, users can set the command-line parameter split_fastq to split the files into 20 million reads each using the SPLIT_FASTQ module.

#### Raw read quality control

Raw read quality is checked separately for each fastq file with the FASTQC module that adopts the FastQC software (Andrews, 2010).

#### Debarcoding, adapter trimming, and mapping

Most generic read alignment tools can only handle genomic reads. Therefore, cell barcodes for tracking cell origins of genomic DNA need to be kept as a part of the sequence identifiers of the corresponding genomic DNA reads (debarcoding). Enabled by ADD_BARCODE_TO_READ, a module integrating the *sinto barcode* command (Stuart et al., 2020), scATACpipe prepends cell barcodes to the original sequence identifiers of corresponding genomic DNA reads (R1 and R3) in the format of @barcode:sequence_identifier.

After debarcoding, scATACpipe adopts the CUTADAPT module, which uses the Cutadapt software (Martin, 2011), for adaptor trimming to improve mapping quality. And the trimmed reads are mapped against the reference genome by BWA_MAP module that calls BWA (Li and Durbin, 2009).

#### BAM file manipulation

The FILTER_BAM module is implemented to remove reads that are improperly mapped or of mapping quality less than 20, reads derived from fragments shorter than 38 bp or longer than 2000 bp, and optionally reads aligned to the organellar genome. As a next step, different lanes of sequencing data (if any) from the same sample in the same library preparation are combined into a single BAM file with the COMBINE_BAM module. Following that, the DEDUP_BAM module first adjusts the mapping coordinates of read pairs with soft clipping (configurable), then collapses read pairs with the same cell barcodes, the same genomic mapping positions at both ends into distinct read pairs. This initial DEDUP_BAM step is required to determine the valid barcodes through the “inflection point” method (See 3.2.1.6 Barcode correction).

#### Barcode correction

Some bases of cell barcodes might be called incorrectly due to PCR and sequencing errors. By providing a whitelist of barcodes, barcode correction can be performed to enhance the accuracy of downstream analysis. scATACpipe provides two options for cell barcode correction. By default, the CORRECT_BARCODE_PHENIQS module is used for probabilistic barcode correction, where frequency of barcodes in the whitelist and base quality are considered to calculate posterior probabilities, and the barcode for a cell is corrected to the barcode in the whitelist with the highest probability above a given cutoff (Galanti et al., 2021). Additionally, the CORRECT_BARCODE module is provided to use a “naïve” algorithm for faster but less accurate barcode correction: barcodes that are in the whitelist remain untouched, and barcodes with a single mismatch to a unique whitelisted barcode are corrected to the whitelist barcode, while barcodes with multiple mismatches are discarded. Furthermore, a helper module called GET_WHITELIST_BARCODE is implemented to determine the appropriate barcode whitelist file from a given folder (assets/whitelist_barcodes) containing candidate barcode whitelist files. If a candidate file has the largest intersection with the unique cell barcodes of a given library and more than 50% of unique cell barcodes of the given library are in the candidate whitelist file, then the candidate whitelist file is considered as the appropriate barcode whitelist file.

The corrected barcodes will then be added into BAM files to the “CB” tag with TAG_BAM module and a final round of duplication removal is performed with DEDUP_BAM2 module according to the “CB” tags and end mapping coordinates.

#### BAM file quality control

The overall alignment quality of each filtered and deduplicated library is checked using the QUALIMAP module, which leverages the Qualimap2 software (Okonechnikov et al., 2016).

#### Fragment file generation

scATACpipe uses GET_FRAGMENTS module to generate coordinate shifted and sorted, block-gzip compressed (bgzip) fragment files (<https://support.10xgenomics.com/single-cell-atac/software/pipelines/latest/output/fragments>) which is compatible with ArchR. Specifically, the start and end coordinates of fragments are shifted by +4 and -5 respectively.

### 10x Genomics Cell Ranger ATAC-based preprocessing modules

Pre-built genome index and annotation files for the human and mouse can be downloaded from 10x Genomics website (<https://support.10xgenomics.com/single-cell-atac/software/downloads/latest>) and provided as a command-line parameter for 10x Genomics Cell Ranger ATAC analysis. The genome sequences for other species or customized human or mouse genomes can be provided and processed as mentioned in Section 3.2.1.1. Subsequently, the CELLRANGER_INDEX module, which calls the cellranger-atac mkref command, is used to build the genome index for mapping. To download the corresponding annotation files, the pipeline uses either the DOWNLOAD_FROM_UCSC_GTF or DOWNLOAD_FROM_ENSEMBL_GTF module and process them with PREP_GTF (see details in Section 3.2.4.1). Alternatively, users can provide reference genome sequences and annotation files via command-line parameters.

The MATCH_SAMPLE_NAME module modifies the names of the fastq files to be compatible with the CELLRANGER_ATAC_COUNT module, which leverages the *cellranger-atac count* command.

The CELLRANGER_ATAC_COUNT module maps and quantifies scATAC-seq reads, as well as performs some basic downstream analyses (https://support.10xgenomics.com/single-cell-atac/software/pipelines/latest/algorithms/overview). Briefly, barcodes are corrected using a Hamming distance algorithm, allowing one mismatch to the whitelist barcodes included in 10x Genomics Cell Ranger ATAC software. The adaptor sequences are trimmed using an algorithm similar to Cutadapt (Martin, 2011). The trimmed reads are then mapped to the specified reference genome using a modified BWA-mem algorithm. After the adjustment for soft clipping at both ends, fragment duplicates are marked based on the start and end positions, and hashed barcodes. Duplicate marked BAM files are further filtered to retain all fragment with a MAPQ > 30 on both reads, non-mitochondrial, not chimerically mapped, and mapped to a primary contig (a gene-containing contig). The deduplicated BAM files are converted to bgzip-compressed fragment files, with the start and end coordinates of fragments shifted by +4 and -5, respectively. The Cell Ranger ATAC analysis-based mode also performs peak calling using aggregate scATAC-seq data, cell calling, peak-by-barcode count matrix generation, dimension reduction by principal component analysis, cell clustering (K-means and graph-based clustering), UMAP/tSNE embedding, peak annotation, TF motif enrichment analysis, and differential accessibility analysis by comparing out-of-cluster TF motif enrichment scores with the in-cluster TF motif enrichment scores. Lastly, fragments from valid cells are prepared into a ArchR compatible bgzipped format using the FILTER_CELL module.

### Chromap-based preprocessing modules

Modules mentioned in Sections 3.2.1.1 are used to prepare genome files. The CHROMAP_INDEX module, a wrapper of the *chromap -i* command, creates Chromap index files. The GET_WHITELIST_CHROMAP module determines the correct barcode whitelist before feeding it into CHROMAP_ATAC for preprocessing, a wrapper of the *chromap --preset atac* command, where *--preset atac* refers to a set of parameters that are tailored specifically for mapping and manipulating ATAC-seq and scATAC-seq reads. Briefly, Chromap is instructed to trim the adapters at the 3' end before mapping the reads with a maximum insert size of 2000. The duplicate reads are then removed at the cell level. This preset will also apply Tn5 shift, where the 5’ end positions of forward and reverse mapping reads are shifted by +4 and -5, respectively. The Chromap algorithm estimates barcode abundance and uses the abundance information to correct barcodes that are one Hamming distance from any whitelist barcode. Then bgzipped fragment files are generated by the FILTER_CELL_CHROMAP module using an inflection point-like method.

# Modules for downstream analysis

scATACpipe takes advantages of the scalability and efficiency of ArchR for downstream analysis of scATAC-seq data. Each of the major functions of the ArchR package is wrapped in an individual Nextflow modules, whose parameters can be set via the module configuration file. ArchR currently only supports scATAC-seq data analyses based on the human reference genome assemblies, hg19 and hg38, and the mouse reference genome assemblies, mm9 and mm10. Given that it is error-prone and time-consuming to apply ArchR to other species or genome assemblies, we developed a set of R functions and modules that facilitate creating custom gene annotation and genome annotation objects, which are two of the inputs required for ArchR-based analyses using any other annotated reference genomes. As a result, scATACpipe enables the streamlined analysis of scATAC-seq data from any species with an annotated reference genome using the algorithms in ArchR.

#### Helper modules

GTF annotation files can be downloaded using the DOWNLOAD_FROM_UCSC_GTF or DOWNLOAD_FROM_ENSEMBL_GTF modules or specified via a command-line parameter. In addition, scATACpipe uses the PREP_GTF module to fix potentially malformed GTF files such as removing scaffold annotations related to alternative haplotypes, prefixing the scaffold names with "chr", and ensuring that chromosome names in the GTF files are consistent with those in the primary reference genome assembly file. For creating gene annotation and genome annotation objects, we implemented the BUILD_BSGENOME, BUILD_TXDB, BUILT_GENE_ANNOTATION, and BUILD_GENOME_ANNOTATION modules. The organellar genome(s) (chrM, and chrPltd), and scaffolds which only contain annotated TSSs within their first and last 2-kb regions are excluded in the process of generating gene annotation and genome annotation objects. The PREP_FRAGMENT module ensures that fragment files are compatible with annotation files.

#### Creating ArchR project and performing cell-level QC

ArchR uses an Arrow file as its base unit for data analysis (Granja et al., 2021). Arrow files are HDF5-backed files stored on hard drives, containing metadata, fragments, and data matrices for a sample. During subsequent analysis, the Arrow file is updated to include additional layers of information. Briefly, every fragment file is converted into an individual ArchR Arrow file through the ARCHR_CREATE_ARROWFILES module, which invokes the createArrowFiles function. Moreover, quality control information (total number of unique nuclear fragments, TSS enrichment score, and fragment size distribution) is calculated for each cell barcode. Additionally, a TileMatrix is created containing insertion counts across genome-wide 500-bp bins, as well as a GeneScoreMatrix containing predicted gene expression based on weighting insertion counts in tiles nearby gene promoters. Cells with low quality can be identified by specifying filterTSS and filterFrags. Because TSS enrichment score depends on the set of TSSs used and the type of cell, the filterTSS cutoff should be set with caution.

After Arrow file creation, doublet (a single droplet that contains a single barcoded bead with more than one nucleus) scores can be calculated and added to the Arrow file. To accomplish this, we implemented ARCHR_ADD_DOUBLETSCORES module, a wrapper of the addDoubletScores function in ArchR for calculating doublet scores. In addition, we implemented AMULET_DETECT_DOUBLETS module to determine doublets/multiples with AMULET (Thibodeau et al., 2021).

Through the ARCHR_ARCHRPROJECT module, which calls the ArchRProject function, an ArchRProject object is created to integrate multiple Arrow files into a single R object. ArchRProject is a small object that is stored in memory. ARCHR_ARCHRPROJECT_QC generates plots showing distribution of TSS enrichment scores, number of unique nuclear fragments, and fragment sizes using various methods for cell-level QC. The ARCHR_FILTER_DOUBLETS module that calls the filterDoublets function can be used to remove doublets when doublet scores are added using the built-in method of ArchR. Alternatively, AMULET_FILTER_DOUBLETS can be used to remove doublets if doublets are determined by AMULET.

#### Dimension reduction and batch correction

Utilizing the ARCHR_DIMENSION_REDUCTION module, which calls the addIterativeLSI function, iterative latent semantic indexing (LSI)-based dimension reduction is performed on the TileMatrix. It is necessary to adjust for batch effects when multiple samples are included in the ArchRProject. ArchR uses the fast, sensitive, and accurate Harmony algorithm (Korsunsky et al., 2019) to correct for batch effects. Batch effects are corrected using the ARCHR_BATCH_CORRECTION module, which invokes the addHarmony function after iterative LSI dimension reduction.

#### Clustering and embedding

Seurat (Butler et al., 2018) and Scran (Lun et al., 2016) are two graph-based clustering methods incorporated into the ArchR package. scATACpipe adopts Seurat out of the consideration of execution speed. Under the reduced dimension sub-space, cells are clustered using the ARCHR_CLUSTERING module, which calls the addClusters function. Clusters of cells are further projected into a two-dimensional space for easy visualization using the ARCHR_EMBEDDING module, which calls the addUMAP and addTSNE functions.

#### Finding the cluster identity

Without any matched scRNA-seq dataset, the ARCHR_MARKER_GENE module, a wrapper of the getMarkerFeatures and getMarkers functions, is employed to determine marker genes with the predicted scores of genes (GeneScoreMatrix), and these markers are then used to infer a cluster’s identity. In addition, this module can be used to visualize marker genes as heatmaps, embedding plots, and genome track plots.

With matched scRNA-seq data provided, the ARCHR_SCRNASEQ_UNCONSTRAINED module is used for an initial round of unconstrained integration of the scATAC-seq data and the scRNA-seq data. Using the resulting mapping information between clusters of the scATAC-seq cells and those of the scRNA-seq cells, the ARCHR_SCRNASEQ_CONSTRAINED module is used for a second round of refined, constrained integration. Further annotation of cluster identity is performed using pseudo-scRNA-seq gene expression profiles (GeneIntegrationMatrix) that result from the unconstrained or preferably the constrained integration with scRNA-seq data.

With matched scRNA-seq data provided for integrated analysis, additional downstream analysis will be performed separately on data determined by scATAC-seq data only (“clusters”) and data undergoing unconstrained/constrained integration (“cluster2”).

#### Generating pseudo-bulk replicates

For subsequent integrative analyses, such as peak calling, co-accessibility analysis, and peak-to-gene linkage analysis, replicate measurements are required to obtain statistical significance. The scATAC-seq data, however, is too sparse to perform this analysis using individual cells as replicates. To overcome the sparsity, Modules ARCHR_PSEUDO_BULK_CLUSTERS and ARCHR_PSEUDO_BULK_CLUSTERS2 subsample and merge multiple cells from each group so that the combined data resembles bulk ATAC-seq data with each pseudo-sample regarded as a pseudo-bulk replicate. ArchR creates multiple pseudo-bulk replicates for each cell group. These modules leverage the addGroupCoverage function.

#### Calling peaks

The ARCHR_CALL_PEAKS_CLUSTERS or ARCHR_CALL_PEAKS_CLUSTERS2 module is used to generate a reproducible MACS2-derived merged peak set, and a matrix of insertion counts for the merged peak set (peakMatrix) which is added to the ArchRProject. These modules call the functions addReproduciblePeakSet and addPeakMatrixuses with a default peak-calling method using MACS2 in ArchR, though a TileMatrix-based peak calling method is available.

#### Identifying marker peaks and pairwise differential peaks

Marker peaks, peaks preferentially belong to a specific cell group, are identified using the ARCHR_GET_MARKER_PEAKS_CLUSTERS or the ARCHR_GET_MARKER_PEAKS_CLUSTERS2 module, which call the getMarkerFeatures and getMarkers functions. By calling the plotMarkerHeatmap and plotMarkers functions, the two modules also generate heatmaps, MA plots, and volcano plots for the visualization of marker peaks. Two other modules ARCHR_MARKER_PEAKS_IN_TRACKS_CLUSTERS and ARCHR_MARKER_PEAKS_IN_TRACKS_CLUSTERS2 are used to generate tracks for visualizing peaks along marker genes, leveraging the plotBrowserTrack function. Differential peaks between two cell clusters are determined by pairwise testing using the ARCHR_PAIRWISE_TEST_CLUSTERS or ARCHR_PAIRWISE_TEST_CLUSTERS2 modules. Additionally, these two modules generate MA plots and volcano plots showing differential peaks using the plotMarkers function.

#### Enrichment analysis of transcription factor binding motif and other features

The ARCHR_MOTIF_ENRICHMENT_CLUSTERS and ARCHR_MOTIF_ENRICHMENT_CLUSTERS2 modules are for the identification of enriched transcription factor (TF) binding motifs or other features, such as ENCODE TF binding sites, bulk ATAC-seq peaks, and ChIP-seq peaks in marker peaks and differential peaks. Internally, these modules call addMotifAnnotations, addArchRAnnotations, or addPeakAnnotations, to create a motif overlap matrix, an interval overlap matrix, or a custom feature overlap matrix. The resulting matrix is used by the peakAnnoEnrichment function to identify enriched motifs or other features. These modules also create scatterplots and heatmaps to display enriched motifs and other features.

#### ChromVAR deviation enrichment analysis

ChromVAR (Schep et al., 2017) is a tool for predicting enrichment of TF activity at single cell level with Tn5 transposase insertion site bias corrected. The ChromVar algorithm is reimplemented in ArchR with improved run time and memory usage. The ARCHR_MOTIF_DEVIATIONS_CLUSTERS and ARCHR_MOTIF_DEVIATIONS_CLUSTERS2 modules in scATACpipe are used to predict the enrichment of TF activity by calling the addDeviationsMatrix and getVarDeviations functions in ArchR. These modules also output scatterplots showing TF motif variability, ridge plots showing z-score profiles of TF motifs with highly variable activity across cell clusters, and embedding plots showing TF motif deviation scores, TF gene scores, and TF pseudo expression when matched scATAC-seq data is integrated with the scATAC-seq data, in each individual cell. Apart from TF motif-based deviation analysis, these two modules can also perform other feature deviation analyses and output similar plots using ArchR-supported region sets or custom region sets.

#### Footprinting analysis

The ARCHR_FOOTPRINTING_CLUSTERS and ARCHR_FOOTPRINTING_CLUSTERS2 modules call the functions getPositions, getFootprints, and plotFootprints functions to calculate and plot aggregate footprints of TFs of interest for each cell cluster. The modules can also determine and plot footprints of transcription start sites (TSSs).

#### Integrative analysis

Co-accessibility among scATAC-seq peaks in low-overlapping aggregates of unique cells are inferred and visualized using the ARCHR_COACCESSIBILITY_CLUSTERS and ARCHR_COACCESSIBILITY_CLUSTERS2 modules, which call the functions addCoaccessibility, getCoaccessibility, and plotBrowserTrack. When matched scRNA-seq data is integrated with the scATAC-seq data, links between peaks and potential target genes can be inferred and visualized using the ARCHR_PEAK2GENELINKAGE_CLUSTERS2 module, which calls the addPeaks2GeneLinks, getPeaks2GeneLinks, and plotBrowserTrack functions. Also generated by this module are side-by-side heatmaps showing the consistency between gene scores and pseudo expression of genes with peaks linked with their promoter peaks. Positive TFs are those whose gene expression (gene scores or pseudo expression) is positively correlated with their motif deviation scores. Positive TFs are determined in scATACpipe using the modules ARCHR_GET_POSITIVE_TF_REGULATOR_CLUSTERS and ARCHR_GET_POSITIVE_TF_REGULATOR_CLUSTERS2 modules, which call the functions getGroupSE and correlateMatrices functions.

#### Trajectory analysis

The ARCHR_TRAJECTORY_CLUSTERS2 module uses the addTrajectory and plotTrajectory functions to determine the trajectory of cells, given a rough clustering order backbone that is derived from matched scRNA-seq data. This module also generates pseudo-time heatmaps showing motif deviation z-scores (MotifMatrix), gene scores (GeneScoreMatrix), integration-derived gene expression (GeneIntegrationMatrix), and peaks (PeakMatrix) by calling the getTrajectory and plotTrajectoryHeatmap functions. Furthermore, this module can perform integrative pseudo-time analysis by calling the correlateTrajectories and plotTrajectoryHeatmap functions.

#### Generating cluster-specific tracks for visualization

To facilitate custom visualization of scATAC-seq, scATACpipe generates cluster-specific tracks in the BED, BAM, or BigWig format. Briefly, the cell barcodes and their clustering information are exported by using the ARCHR_GET_CLUSTERING_TSV module. Cluster-specific BED, BAM, and BigWig files are generated with the cell barcodes and clustering information using a custom SPLIT_BED module, along with a SPLIT_BAM module, which calls the *sinto filterbarcodes* function from sinto and the *bamCoverage* commands from deepTools package.

#### Report-generating modules

To enable an integrative view of the analysis results, scATACpipe utilizes and extends MultiQC (Ewels et al., 2016) to generate an interactive HTML report. When the default preprocessing option is chosen, scATACpipe generates a report by taking results as input from a series of modules: FASTQC, CORRECT_BARCODE/CORRECT_BARCODE_PHENIQS, CUTADAPT, BAM_FILTER, REMOVE_DUPLICATE, QUALITMAP, and ArchR-related modules. When the 10x Genomics Cell Ranger ATAC-based or Chromap-based preprocessing option is chosen, MultiQC produces a report by taking the results from ArchR-related modules, whereas a preprocessing report is included in the output from the CELLRANGER_ATAC_COUNT module.

**References**

Andrews, S. (2010). *FastQC: A Quality Control Tool for High Throughput Sequence Data* [Online]. Available: <http://www.bioinformatics.babraham.ac.uk/projects/fastqc/> [Accessed September 10 2021].

Buenrostro, J.D., Giresi, P.G., Zaba, L.C., Chang, H.Y., and Greenleaf, W.J. (2013). Transposition of native chromatin for fast and sensitive epigenomic profiling of open chromatin, DNA-binding proteins and nucleosome position. *Nature Methods* 10**,** 1213-1218.

Butler, A., Hoffman, P., Smibert, P., Papalexi, E., and Satija, R. (2018). Integrating single-cell transcriptomic data across different conditions, technologies, and species. *Nature Biotechnology* 36**,** 411-420.

Ewels, P., Magnusson, M., Lundin, S., and Käller, M. (2016). MultiQC: summarize analysis results for multiple tools and samples in a single report. *Bioinformatics* 32**,** 3047-3048.

Galanti, L., Shasha, D., and Gunsalus, K.C. (2021). Pheniqs 2.0: accurate, high-performance Bayesian decoding and confidence estimation for combinatorial barcode indexing. *BMC Bioinformatics* 22**,** 359.

Granja, J.M., Corces, M.R., Pierce, S.E., Bagdatli, S.T., Choudhry, H., Chang, H.Y., and Greenleaf, W.J. (2021). ArchR is a scalable software package for integrative single-cell chromatin accessibility analysis. *Nat Genet* 53**,** 403-411.

Karolchik, D., Hinrichs, A.S., and Kent, W.J. (2009). The UCSC Genome Browser. *Current Protocols in Bioinformatics* 28**,** 1.4.1-1.4.26.

Korsunsky, I., Millard, N., Fan, J., Slowikowski, K., Zhang, F., Wei, K., Baglaenko, Y., Brenner, M., Loh, P.-R., and Raychaudhuri, S. (2019). Fast, sensitive and accurate integration of single-cell data with Harmony. *Nature Methods* 16**,** 1289-1296.

Li, H., and Durbin, R. (2009). Fast and accurate short read alignment with Burrows–Wheeler transform. *Bioinformatics* 25**,** 1754-1760.

Lun, A., Mccarthy, D., and Marioni, J. (2016). A step-by-step workflow for low-level analysis of single-cell RNA-seq data with Bioconductor [version 2; peer review: 3 approved, 2 approved with reservations]. *F1000Research* 5.

Martin, M. (2011). Cutadapt removes adapter sequences from high-throughput sequencing reads. *2011* 17**,** 3.

Newman, V., Moore, B., Sparrow, H., and Perry, E. (2018). The Ensembl Genome Browser: Strategies for Accessing Eukaryotic Genome Data. *Methods Mol Biol* 1757**,** 115-139.

Okonechnikov, K., Conesa, A., and García-Alcalde, F. (2016). Qualimap 2: advanced multi-sample quality control for high-throughput sequencing data. *Bioinformatics (Oxford, England)* 32**,** 292-294.

Ou, J., Liu, H., Yu, J., Kelliher, M.A., Castilla, L.H., Lawson, N.D., and Zhu, L.J. (2018). ATACseqQC: a Bioconductor package for post-alignment quality assessment of ATAC-seq data. *BMC Genomics* 19**,** 169.

Schep, A.N., Wu, B., Buenrostro, J.D., and Greenleaf, W.J. (2017). chromVAR: inferring transcription-factor-associated accessibility from single-cell epigenomic data. *Nat Methods* 14**,** 975-978.

Stuart, T., Srivastava, A., Lareau, C., and Satija, R. (2020). Multimodal single-cell chromatin analysis with Signac. *bioRxiv***,** 2020.2011.2009.373613.

Thibodeau, A., Eroglu, A., Mcginnis, C.S., Lawlor, N., Nehar-Belaid, D., Kursawe, R., Marches, R., Conrad, D.N., Kuchel, G.A., Gartner, Z.J., Banchereau, J., Stitzel, M.L., Cicek, A.E., and Ucar, D. (2021). AMULET: a novel read count-based method for effective multiplet detection from single nucleus ATAC-seq data. *Genome Biol* 22**,** 252.
