# Supplementary figures and images for: scATACpipe: A nextflow pipeline for comprehensive and reproducible analyses of single cell ATAC-seq data
Source: Front Cell Dev Biol. 2022 Sep 27;10:981859. doi: 10.3389/fcell.2022.981859 (PMC9551270; doi:10.3389/fcell.2022.981859)

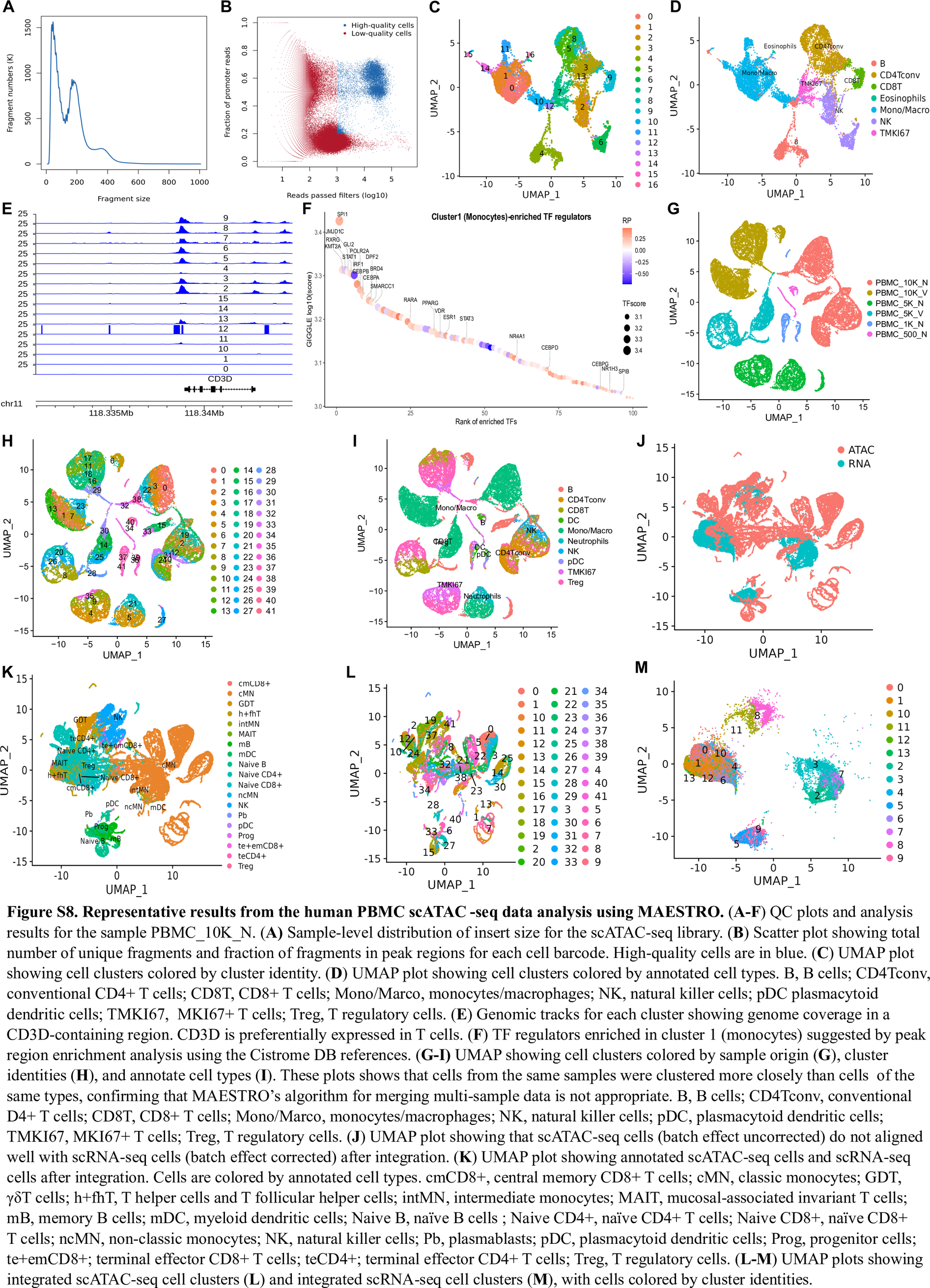

Supplement: Supplementary file 1 [file DataSheet1.ZIP › supplementary/Figure S8.tif]

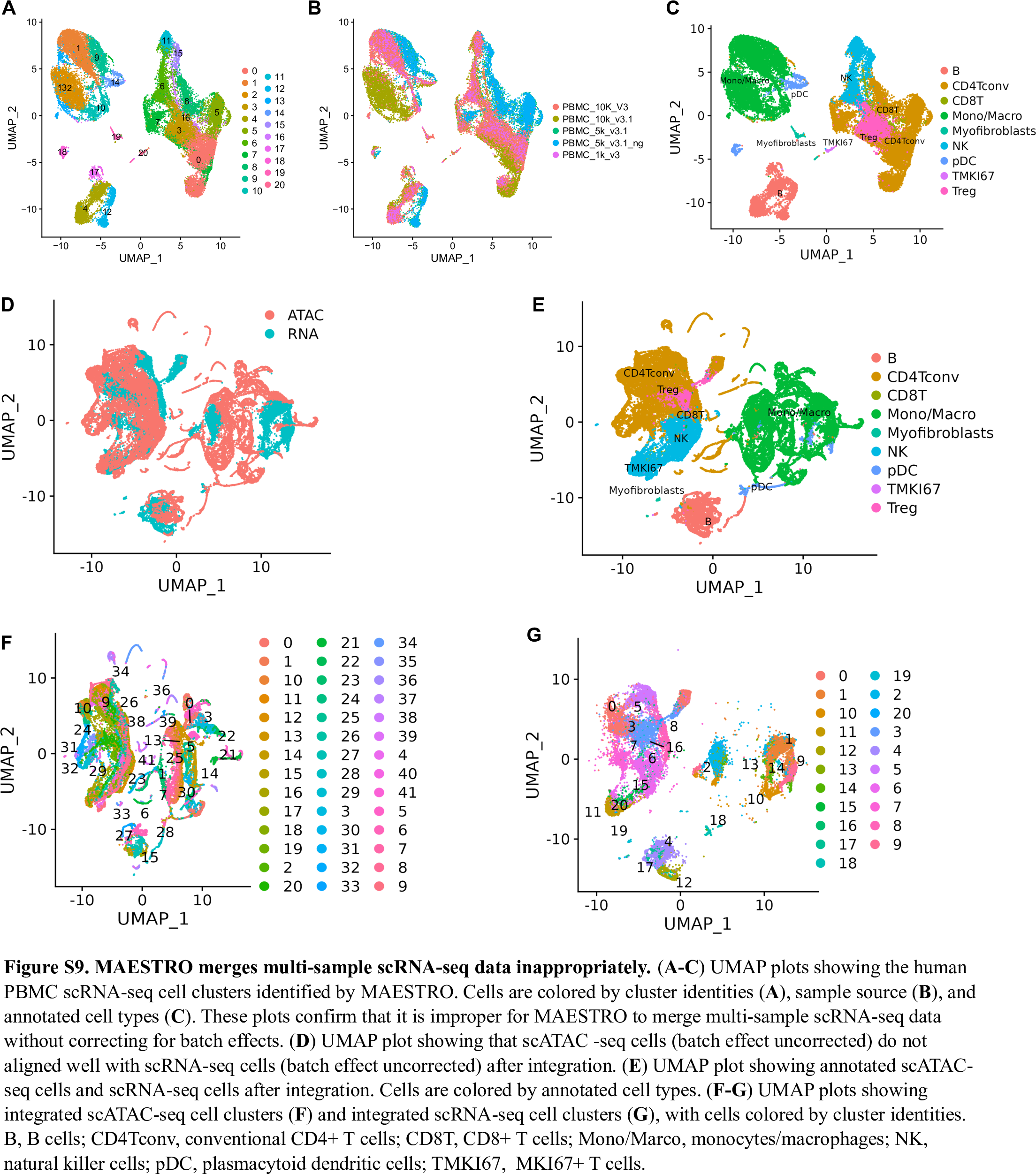

Supplement: Supplementary file 1 [file DataSheet1.ZIP › supplementary/Figure S9.tif]

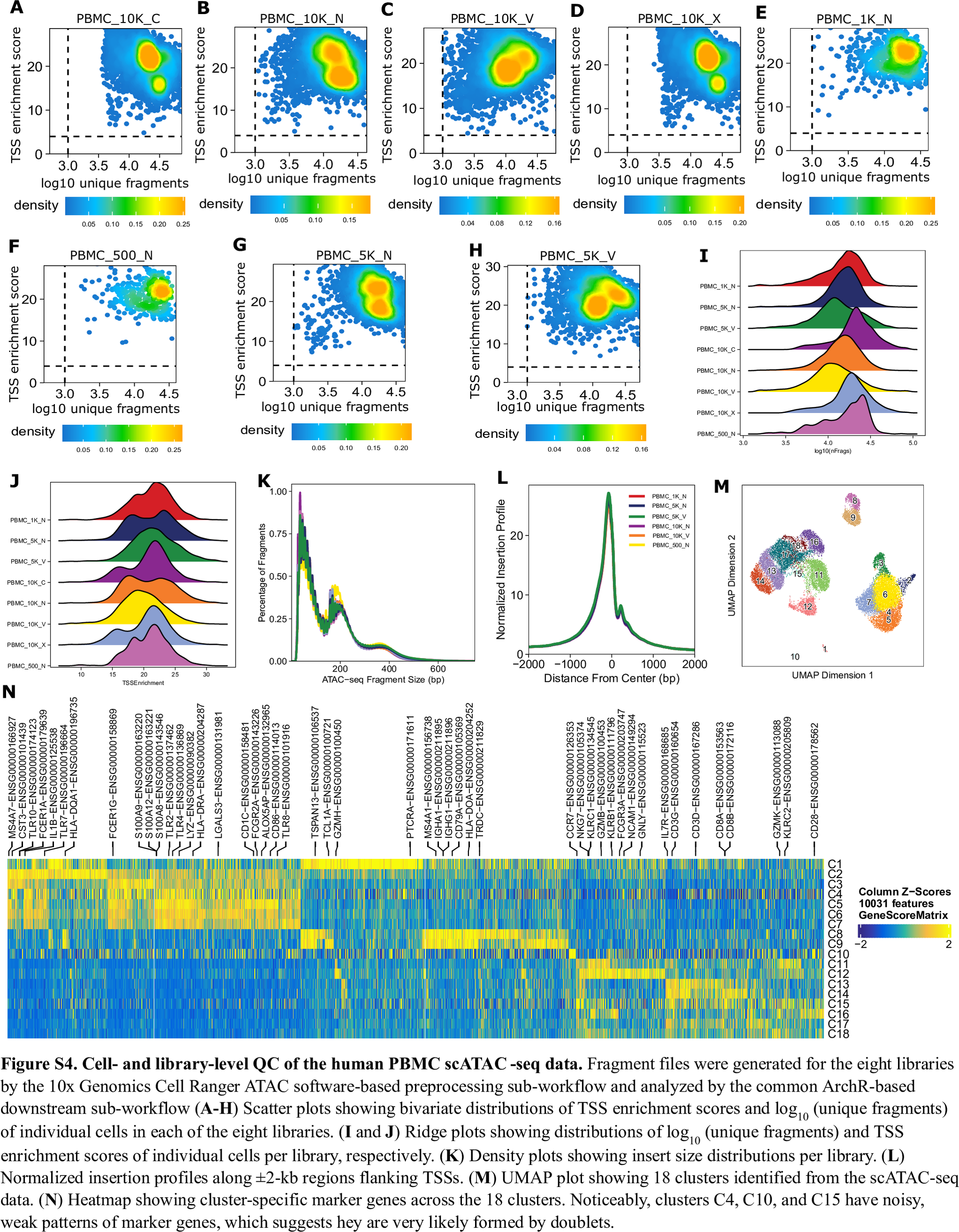

Supplement: Supplementary file 1 [file DataSheet1.ZIP › supplementary/Figure S4.tif]

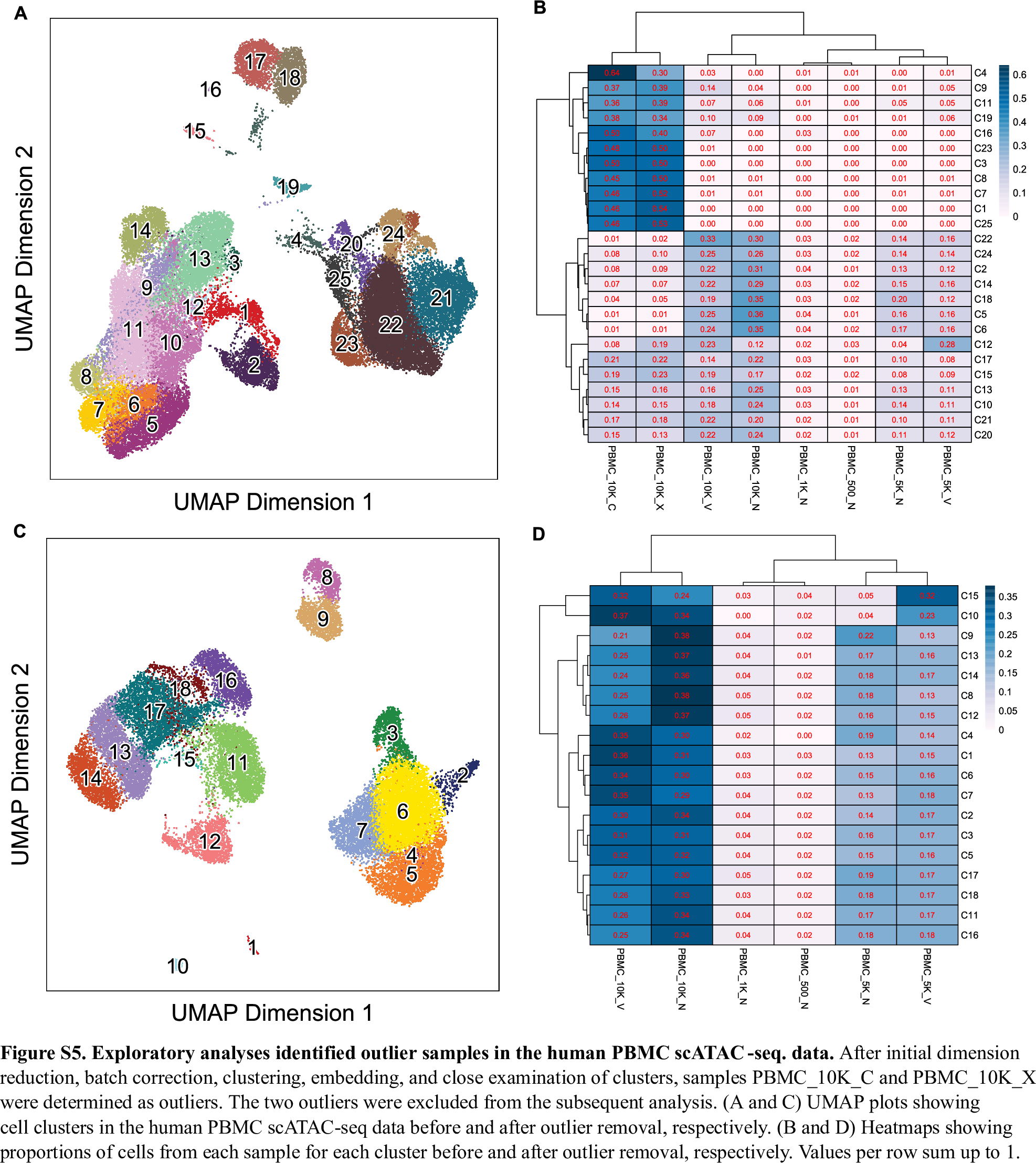

Supplement: Supplementary file 1 [file DataSheet1.ZIP › supplementary/Figure S5.tif]

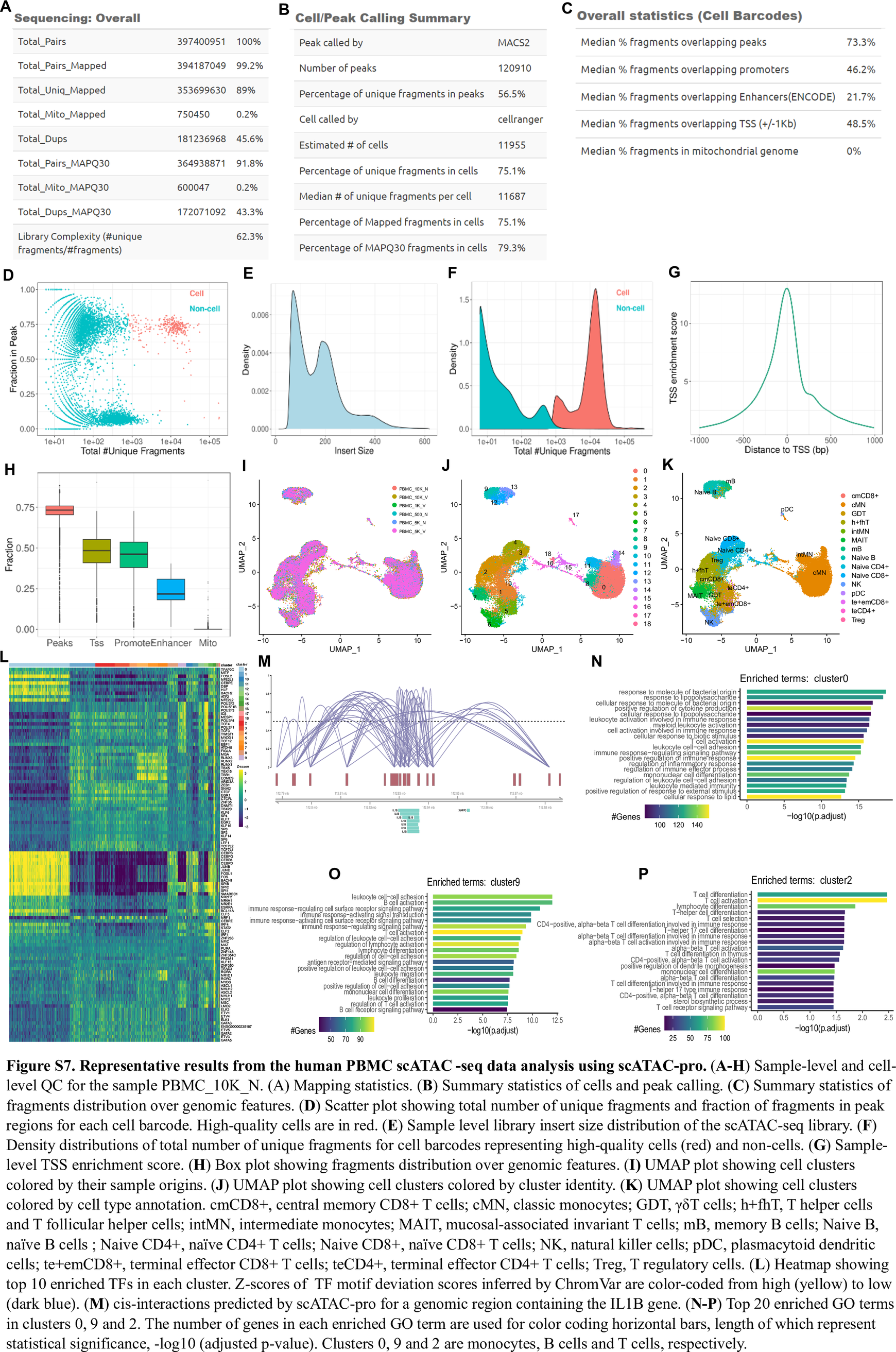

Supplement: Supplementary file 1 [file DataSheet1.ZIP › supplementary/Figure S7.tif]

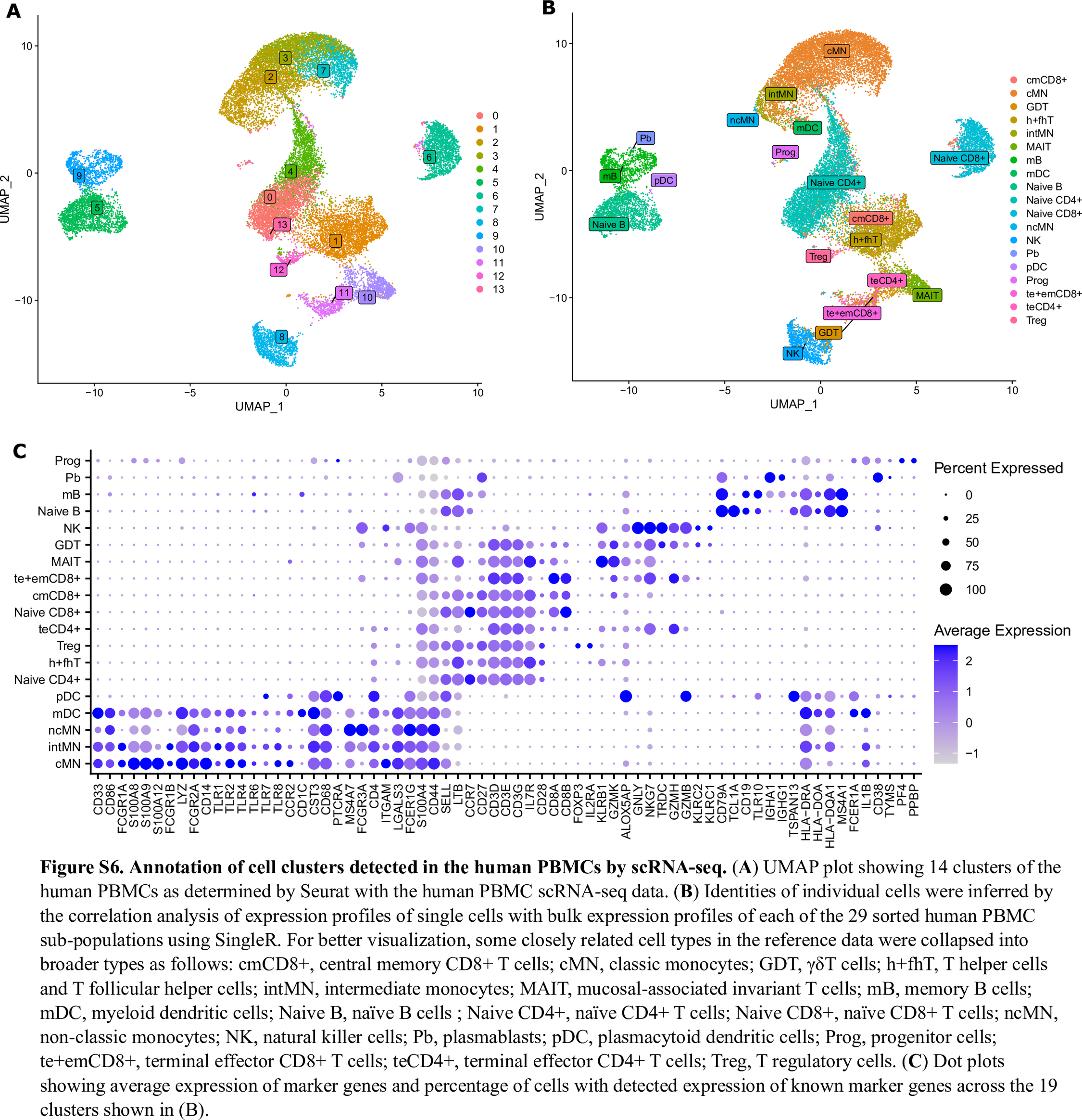

Supplement: Supplementary file 1 [file DataSheet1.ZIP › supplementary/Figure S6.tif]

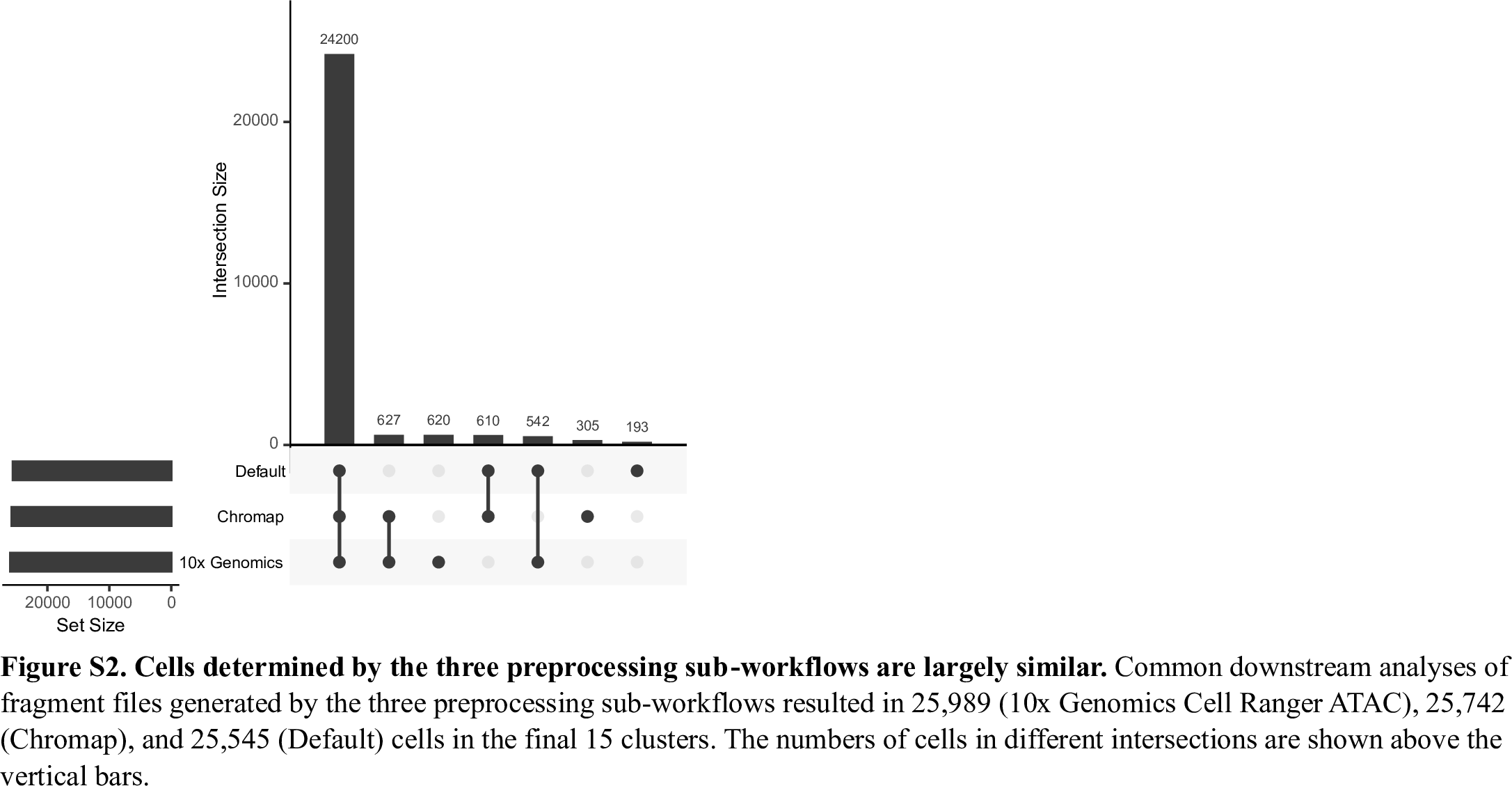

Supplement: Supplementary file 1 [file DataSheet1.ZIP › supplementary/Figure S2.tif]

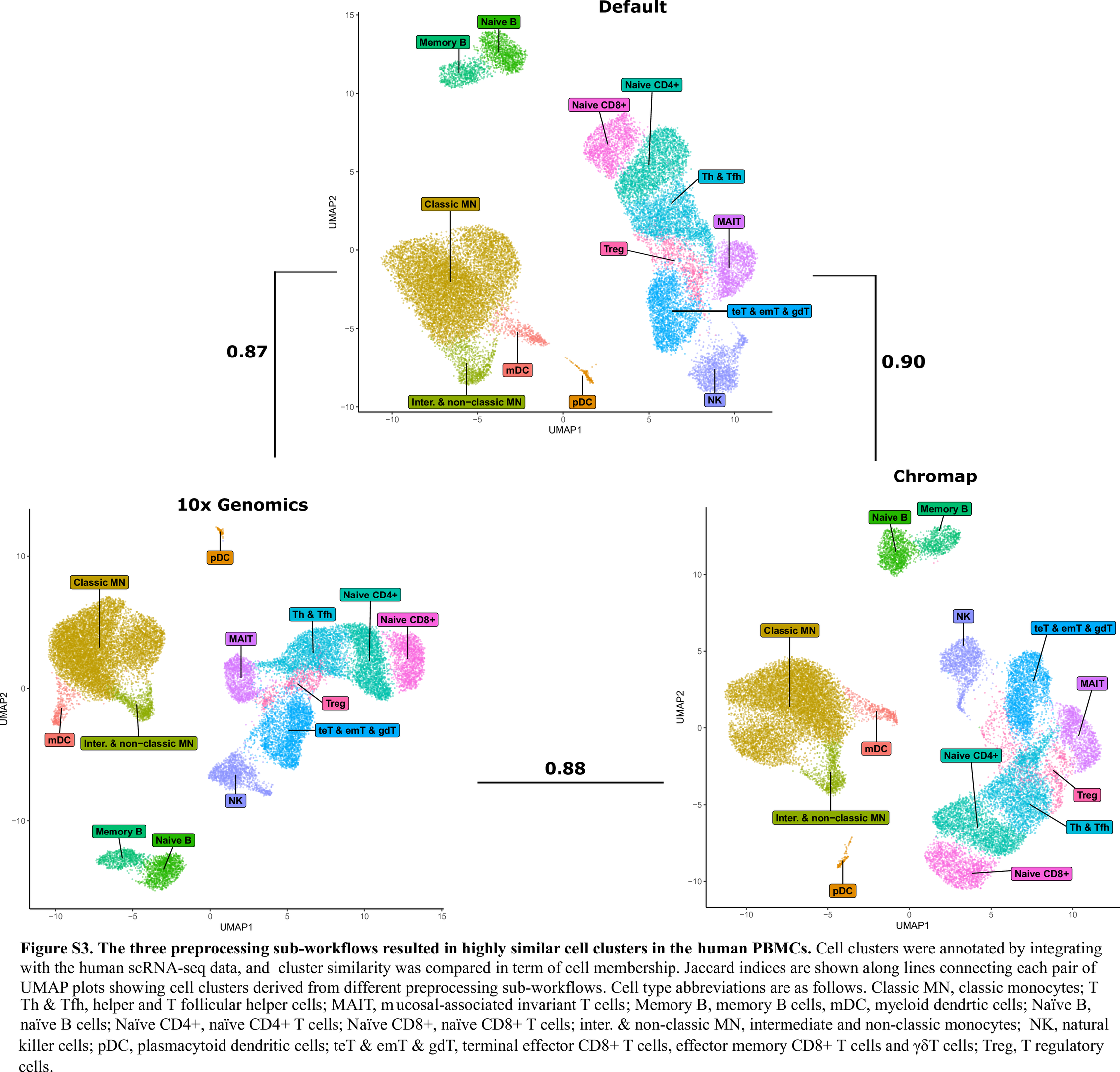

Supplement: Supplementary file 1 [file DataSheet1.ZIP › supplementary/Figure S3.tif]

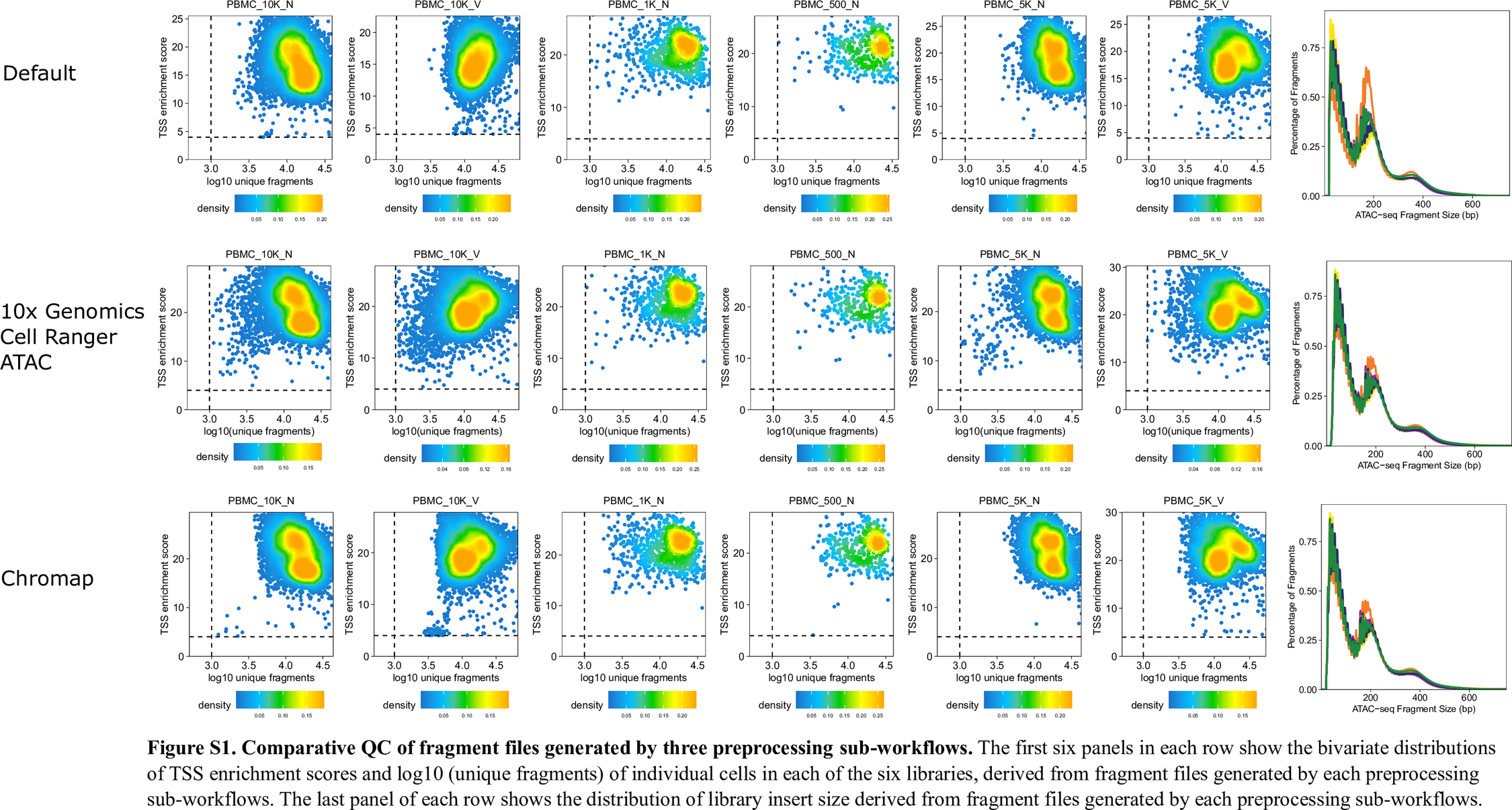

Supplement: Supplementary file 1 [file DataSheet1.ZIP › supplementary/Figure S1.tif]
